# Transcriptome of iPSC-derived neuronal cells reveals a module of co-expressed genes consistently associated with autism spectrum disorder

**DOI:** 10.1038/s41380-020-0669-9

**Published:** 2020-02-14

**Authors:** K. Griesi-Oliveira, M. S. Fogo, B. G. G. Pinto, A. Y. Alves, A. M. Suzuki, A. G. Morales, S. Ezquina, O. J. Sosa, G. J. Sutton, D. Y. Sunaga-Franze, A. P. Bueno, G. Seabra, L. Sardinha, S. S. Costa, C. Rosenberg, E. C. Zachi, A. L. Sertie, D. Martins-de-Souza, E. M. Reis, I. Voineagu, M. R. Passos-Bueno

**Affiliations:** 1 Hospital Israelita Albert Einstein, São Paulo, Brazil; 2 Departamento de Genética e Biologia Evolutiva, Instituto de Biociências, Universidade de São Paulo, São Paulo, Brazil; 3 Programa Interunidades de Pós-Graduação em Bioinformática, Universidade de São Paulo, São Paulo, Brazil; 4 School of Biotechnology and Biomolecular Sciences, University of New South Wales, Sydney, Australia; 5 Max Delbrück Center for Molecular Medicine in the Helmholtz Association, Berlin, Germany; 6 Laboratory of Neuroproteomics, Department of Biochemistry and Tissue Biology, Institute of Biology, University of Campinas (UNICAMP), São Paulo, Brazil; 7 Núcleo de Neurociências e Comportamento, Departamento de Psicologia Experimental, Instituto de Psicologia, Universidade de São Paulo, São Paulo, Brazil; 8 Instituto Nacional de Biomarcadores em Neuropsiquiatria (INBION), Conselho Nacional de Desenvolvimento Científico e Tecnológico (CNPq), São Paulo, Brazil; 9 Experimental Medicine Research Cluster (EMC), University of Campinas, Campinas, Brazil; 10 Departamento de Bioquímica, Instituto de Química, Universidade de São Paulo, São Paulo, Brazil

**Keywords:** Cell biology, Biological techniques, Genetics, Molecular biology, Neuroscience

## Abstract

Evaluation of expression profile in autism spectrum disorder (ASD) patients is an important approach to understand possible similar functional consequences that may underlie disease pathophysiology regardless of its genetic heterogeneity. Induced pluripotent stem cell (iPSC)-derived neuronal models have been useful to explore this question, but larger cohorts and different ASD endophenotypes still need to be investigated. Moreover, whether changes seen in this in vitro model reflect previous findings in ASD postmortem brains and how consistent they are across the studies remain underexplored questions. We examined the transcriptome of iPSC-derived neuronal cells from a normocephalic ASD cohort composed mostly of high-functioning individuals and from non-ASD individuals. ASD patients presented expression dysregulation of a module of co-expressed genes involved in protein synthesis in neuronal progenitor cells (NPC), and a module of genes related to synapse/neurotransmission and a module related to translation in neurons. Proteomic analysis in NPC revealed potential molecular links between the modules dysregulated in NPC and in neurons. Remarkably, the comparison of our results to a series of transcriptome studies revealed that the module related to synapse has been consistently found as upregulated in iPSC-derived neurons—which has an expression profile more closely related to fetal brain—while downregulated in postmortem brain tissue, indicating a reliable association of this network to the disease and suggesting that its dysregulation might occur in different directions across development in ASD individuals. Therefore, the expression pattern of this network might be used as biomarker for ASD and should be experimentally explored as a therapeutic target.

## Introduction

It has been long recognized that genetic factors play an important role in autism spectrum disorder (ASD) [1, 2], a neurodevelopmental disorder characterized by impairments in social-communicative skills and repetitive behaviors, that affects at least 1% of the population [3, 4]. Although the knowledge regarding genetic risk factors for ASD has greatly improved in recent years [5], mainly driven by the development of high-throughput genomic screening approaches, the complex genetic architecture and the heterogeneity of etiological factors underlying the disorder hamper the understanding of its pathophysiology, the establishment of molecular diagnostic criteria, and the identification of potential therapeutic targets. On the other hand, despite the large number of ASD-candidate genes described so far, they seem to converge on a few final common effectors or molecular pathways [6], suggesting that the different genetic variants associated with the disease may lead to similar functional consequences, which might be reflected in the transcriptional level, protein level or, lately, in the regulation of specific cellular mechanisms.

Based on this premise, whole transcriptome studies have been conducted to explore differentially expressed gene profiles associated with ASD, mainly using postmortem brain tissue from autistic individuals [7–10]. Although consistent results have been found across some of these studies, such as expression alterations of genes related to immune response, neurotransmission and neurodevelopment, it has to be considered that the child/adult brain may not capture dysregulated gene expression associated with ASD that are only transiently present during prenatal development. In this regard, the research on the pathogenesis of neurodevelopmental disorders, including ASD, has benefited from the cellular reprogramming technique. This approach has enabled the generation of neural progenitor cells (NPC) and neurons from ASD patients, therefore producing in vitro models that might recapitulate the features of the developing brain in vivo [11–15]. Some studies using iPSC-derived neuronal cells have explored transcriptome profiles of relatively small samples of idiopathic ASD individuals, including two studies that have selected only macrocephalic patients [16, 17], who represent less than 20% of the ASD patients [18], and a study on a sample enriched with low-functioning normocephalic patients [19]. Considering the genetic and phenotypic heterogeneity of the disease, further studies are needed to increase the number of examined patients, specially to investigate other ASD endophenotypes, and to clarify the correlation between the results found in postmortem brain tissue and those found using in vitro neuronal models.

Here, we generated iPSC lineages from stem cells derived from exfoliated deciduous teeth (SHED) of a cohort of ASD patients enriched with high-functioning individuals to investigate the transcriptional profiles of iPSC-derived neuronal cells at two stages of development: at progenitor state and after complete differentiation to post-mitotic neurons. We examined which temporal window of brain development best fits our in vitro model by comparing the transcriptome profile of the iPSC-derived neural cells with expression profiles of fetal brains at different stages of development and also to adult postmortem brains. Differences between ASD and control samples were addressed both on a single gene and on a systemic level, using gene co-expression network analysis. Comparison of our results with previous transcriptome studies using both iPSC-derived neuronal cells and postmortem brain tissue revealed the consistent dysregulation of a module of synaptic molecules, which may represent a subset of genes exhibiting a pattern of expression that could be used as a biomarker for ASD.

## Materials and methods

### Patients ascertainment

All ASD individuals enrolled in this study (*n* = 6) were males and negative for Fragile-X Syndrome, including five high-functioning patients and one low-functioning patient (IQ < 70; Supplementary Table S1). The control sample consisted of 6 male individuals with no history of ASD or any other neurodevelopmental disorder diagnosis. This project has been approved by the Ethics Committee of the Instituto de Biociências – Universidade de São Paulo (protocol number 1.133.486).

### array-CGH and exome sequencing

The presence of CNVs was evaluated in peripheral blood DNA from patients and their parents by comparative genomic hybridization array (CGH-array) using an Agilent 4X 180K chip (Agilent Technologies, CA, USA). Exome-sequencing libraries were generated using Nextera Rapid Capture Exome kit (Illumina, CA, USA) and sequenced on an Illumina HiSeq 2500 equipment. Reported variants were selected based on a pipeline described in Supplementary Material.

### iPSC generation and characterization

iPSCs lines were generated from stem cells from human exfoliated deciduous teeth (SHED) through retroviral transduction of vectors containing *SOX2, c-Myc, OCT4*, and *KLF4* as described in Griesi-Oliveira et al. [14]. Two to three clones were generated from each sample, which were tested for pluripotency markers expression and for endoderm, mesoderm and ectoderm markers expression after random differentiation (Supplementary Fig. S1; Supplementary Material).

### Neuronal differentiation

Neuronal progenitor cells (NPC) were differentiated from iPSC as described in Griesi-Oliveira et al. [14], and maintained in 0.5x NB media—DMEM/F12 (Thermo Fisher Scientific, CA, USA), plus 0.5x N2-supplement (Thermo Fisher Scientific), and 0.5x B27 serum (Thermo Fisher Scientific)—supplemented with 20 ng/ml of FGF and EGF (Peprotech, NJ, USA; Supplementary Fig. S1A). Some NPC lineages were transduced with SYN::EGFP lenti-virus vector, a vector containing the sequence of green fluorescent protein under the control of the Synapsin gene promoter (kindly donated by Dr. Alysson Muotri). Neurons were obtained after 4 weeks of differentiation from NPC (Supplementary Fig. S1A), using 1x NB media, supplemented with 1 μM of retinoic acid (Sigma-Aldrich, MI, USA). This protocol has been previously proven to generate neurons with active electrophysiological properties [14]. GFP-expressing neurons were sorted out from heterogeneous populations of differentiated neuronal cells by fluorescence-activated cell sorting in a FACSAria II Machine (BD Biosciences, NJ, USA; Supplementary Fig. S2).

### Immunocytochemistry

Cells were fixed and then incubated overnight with one of the primary antibodies: anti-SOX2 (1:100, Millipore, MA, USA; cat# AB5603), anti-Nestin (1:250, Millipore; cat# MAB5326), anti-CTIP2 (1:500, Millipore; cat# ab187668), anti-MAP2 (1:500, Millipore; cat# MAB3418), anti-GFP (Millipore, 1:1000; cat# ab1218). The cells were then stained with Alexa Fluor 488 donkey or Alexa Fluor 594 donkey anti-mouse (Thermo Fisher Scientific, cat# A21202 and A21203, respectively) and visualized with Olympus IX51 confocal microscope (Olympus, Japan).

### RNA sequencing and normalization of expression data

RNA samples from NPC (29 cell lines), from heterogeneous populations of neuronal cells (16 cell lines) or sorted GFP-expressing neurons (7 cell lines) were used for library preparation using the TruSeq RNA Sample Preparation kit (Illumina, CA, USA) and then sequenced on a HiSeq 2500 (Illumina) to generate 100 bp paired-end sequences. Sequences were aligned to the reference human genome (hg19) using TopHat2 [20] and read counts per gene were summarized using HTSeq [21]. Data are available at Gene Expression Omnibus website under the accession number GSE142670. After neuronal proportion estimation by a deconvolution method [22] (Supplementary Material), four neuronal samples with a calculated proportion of neurons lower than 51% were excluded (Supplementary Table S2). Only genes with a Fragments per million kilobase (FPKM) ≥ 1 in more than half of the samples (calculated separately for NPC and neurons) were considered as expressed and retained for analysis (NPC: 13818 genes; neurons: 15026 genes). Expression data were normalized using RUVseq [23] in order to correct for different proportions of neurons within each sample. We checked for the best parameters (control genes and number of co-variates) that would lead to the loss of such correlation (Supplementary Table S3), while leading to the best clustering of replicates (Supplementary Figure S3; see Supplementary Methods).

### Prediction of regional identity and developmental period

To estimate the maturity of the cells and their regional identity, transcriptome profiles of each of NPC and neuron samples were compared with the transcriptome data of brain samples available at BrainSpan Atlas [24] (www.brainspan.org), whose ages vary between 4 post-conceptional weeks until 60 years old, using a machine-learning approach developed by Stein et al. [25] (https://context.semel.ucla.edu/).

### Differential expression and weighted-gene correlation network analysis

Differentially expressed genes were identified using *dream* statistical model available within variancePartition package [26], considering a significance level threshold of FDR ≤ 0.05. Weighted-gene co-expression network analysis was performed separately for NPC and Neurons using WGCNA package from R [27] (Supplementary Material). For each module, gene expression levels of each sample were summarized in an eigengene value which was then used to assess the correlation of a module to disease status, the batch of library preparation or the neuronal proportion within the samples. Functional annotation analysis was performed using the Database for Annotation, Visualization and Integrated Discovery 6.8 (DAVID - https://david-d.ncifcrf.gov/), Ingenuity Pathway Analysis Software (http://www.ingenuity.com/) and/or cameraPR function from limma package [28]. Protein–protein enrichment test was performed using STRING (https://string-db.org/cgi/input.pl). Module-preservation analysis [29] was conducted using *modulePreservation* function from WGCNA, comparing our data with BrainSpan fetal brain samples. Data and analysis code are available at https://github.com/griesik/ASDiPSCTranscriptome.

### Neuronal morphological analysis

Twenty thousand cell-sorted GFP-expressing neurons were plated with 50,000 cells of a heterogeneous population of neuronal cells differentiated from NPC not transduced with SYN::EGFP either from the same individual (non-mixed condition) or from an individual from the opposite group (mixed condition), in wells of an 8-well chamber slide. Seventy-two hours after plating, cells were fixed and immunostained for green fluorescent protein, as described above. Images were taken at ×20 with InCell Analyzer 2200 microscope (GE Healthcare, Chicago IL, USA) and morphological analysis was conducted using Neurphology plugin from ImageJ. Measures obtained were normalized by the number of nuclei in each image and statistical analysis was performed using geepack package from R [30].

### Mass spectrometry-based proteomics

Protein was extracted from NPC of 6 clones (3 patients, 3 controls) and injected into two-dimensional, reverse-phase liquid chromatography using an Acquity UPLC M-Class System (Waters Corporation, MA, USA) coupled to a Synapt G2-Si mass spectrometer (Waters Corporation). Mass spectrometric raw data was processed with Progenesis®QI version 2.1 (Waters Corporation) and proteins were identified. Quantitative data were processed using dedicated algorithms and searched against the Uniprot human proteomics database (https://www.uniprot.org/proteomes/), with the default parameters for ion accounting and quantitation as described in Cassoli et al. [31].

### Variants and Sfari genes enrichment analysis

A list of rare de novo exonic variants found in ASD individuals and their siblings was obtained from Kosmick et al. [32] (a compilation of the original papers from de Rubeis et al. [33]; Iossifov et al. [34]). ASD genes were compiled from Sfari [35] database (as of November 2019) and a list of ID genes was obtained from ID genetics [36] (http://www.ccgenomics.cn/IDGenetics/index.php), as of January 2019 (see Supplementary Methods). Only the protein-coding genes from the co-expression modules were considered for gene set enrichment, which was performed using a two-sided Fisher’s exact test calculated by the R function *fisher.test*, corrected for false discovery rate.

### Module overlap analysis

The function *userListEnrichment* from WGCNA was used to evaluate the overlap between the modules identified in our study to modules identified by others [8–10, 16, 17, 19, 37–39]. For identification of a list of genes with strong evidence of being part of the module of synapse genes associated with ASD, we considered all the modules from the studies that used either fetal/neonatal brain samples or iPSC-derived neurons that presented a significant overlap with M_Neu_1-turquoise, except for the study from DeRosa et al. [19] (see Supplementary Methods).

## Results

### Genetic characterization of ASD patients

DNA from peripheral blood samples from all the patients included in this study was submitted to array-CGH and whole-exome sequencing. We identified two individuals carrying more than one ASD pathogenic variant, suggestive of an oligogenic model of inheritance. Patient F2613 harbors a 345 kb de novo duplication in 17p13.3, which involves six different genes: *ABR, BHLHA9, TUSC5, YWHAE, CRK, MYO1C*. Duplications in this region are well described in the literature [40], and although associated with variable phenotypes and incomplete penetrance, a considerable number of individuals with this copy number variation (CNV) present ASD. This patient also carries a de novo missense variant predicted to be damaging (CADD ≥ 20) in *DOCK1* (NM_001290223: c.4838C>T (p.Pro1613Leu)) [41] an ASD-candidate gene (Sfari score = 3). The other patient, F2688, is a compound heterozygote for rare potentially deleterious missense variants (NM_005045.3: c.7538C>G (p.Ser2513Cys) and c.7634C>T (p.Ala2545Val)) inherited from his parents in the Reelin gene (*RELN*), which is considered a high confidence ASD-candidate (Sfari = 1). These variants lead to reduced secretion of Reelin and impaired Reelin signal transduction in iPSC-derived NPC of this patient, as we have recently described [42]. This patient also harbors a rare de novo loss-of-function (LoF) mutation in another ASD-candidate gene, *CACNA1H* (Sfari score = 2), which affects a splicing site in exon 13 of this gene. Rare de novo LoF mutations in brain expressed genes considered to be LoF-intolerant (pLI ≥ 0.8, as described in ExAC database, http://exac.broadinstitute.org/) or de novo missense variants with CADD-score ≥ 20 were not identified in any of the other four remaining patients. On the other hand, inherited rare potentially damaging (CADD ≥ 20) missense variants were identified in Sfari genes in all the patients. A full description of this genetic characterization is available in Supplementary Table S4.

### iPSC-derived neuronal cells have an expression profile similar to fetal brains at a developmental period important to ASD pathophysiology

**Fig. 1:**
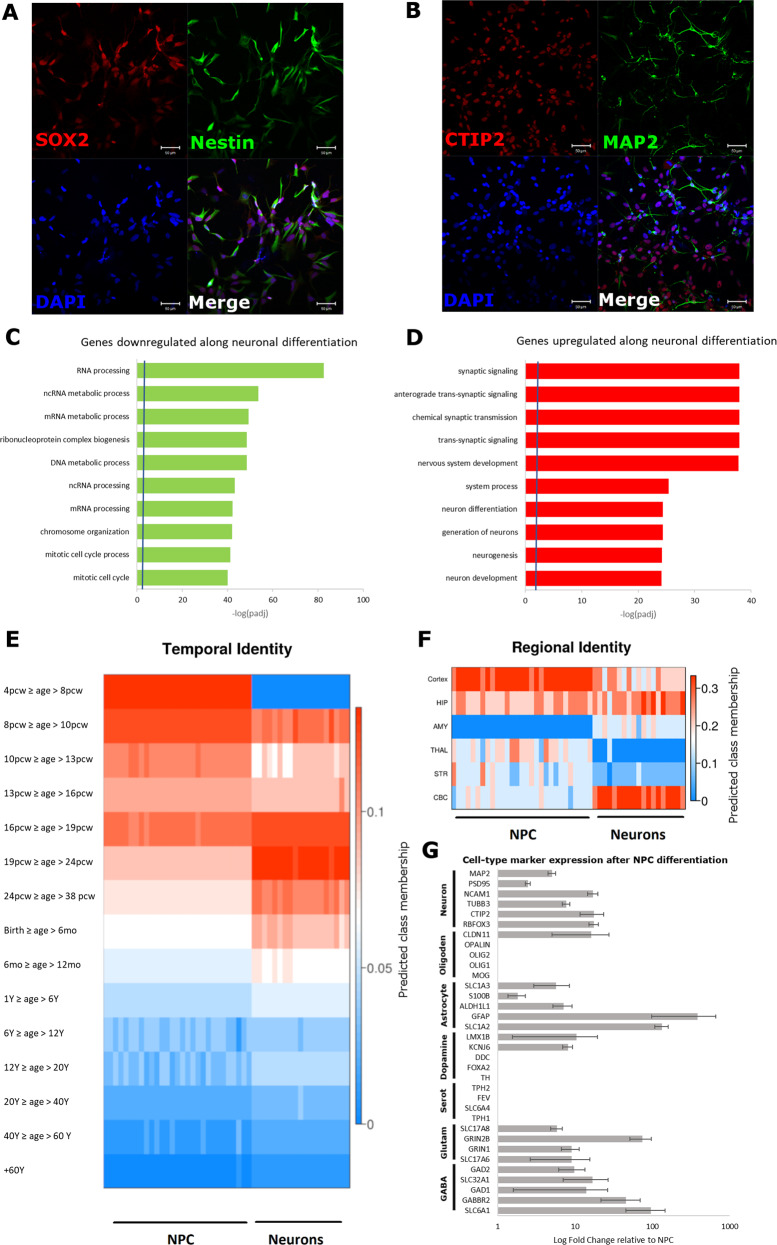
Characterization of neuronal cells derived from ASD and control iPSC lines. **a**, **b** iPSC-derived neuronal cells show expression of typical markers for progenitor cells, such as SOX2 and Nestin, and for neurons, such as MAP2 and CTIP2. **c**, **d** Differential expression analysis reveal that genes downregulated upon differentiation of NPC into neurons are related to cell cycle and cell division, while genes related to neuronal differentiation and function are upregulated. For this analysis all control and ASD samples were used. **e**, **f** Temporal and regional identity of iPSC-derived neuronal cells as predicted by the machine-learning algorithm CoNTExT: NPC display a transcriptome profile similar to in vivo fetal brain samples at 4–10 post-conceptional weeks (pcw), while neurons best match the 16–24 pcw temporal window, evidencing a clear temporal progression in the maturation of the obtained cells. **g** Average expression of select cell-type markers for neurons, astrocytes and oligodendrocytes as well as for glutamatergic, gabaergic, serotoninergic, and dopaminergic neurons.

In order to characterize the neuronal cells obtained from ASD and control iPSC lines using our differentiation protocol, we conducted several analyses. First, we showed that NPC express Nestin and SOX2, while neurons express CTIP2 and MAP2, which are typical cell markers for these cell types (Fig. 1a, b, g). Differentiation protocols may not produce pure populations of the cells of interest and, therefore, the proportion of cells of different types may vary across samples, leading to biased results in gene expression profiling studies [43, 44]. Indeed, we found that the first component of the expression profile of each sample was strongly correlated with the proportion of neurons within the sample before normalization (Supplementary Figure S3).

Once transcriptome data were corrected for this bias (see the Materials and methods section), we first conducted a differential expression analysis between NPC and neurons (using all controls and ASD cells), which revealed that, as expected, genes that were downregulated in neurons after differentiation from NPC are mainly involved in cell cycle and cell proliferation, while upregulated genes are related to the process of neurogenesis (Fig. 1c, d; Supplementary Table S5). We then used CoNTExT framework [25], a machine-learning-based algorithm trained on transcriptome data from BrainSpan Atlas [24], to estimate the stage of brain development and the brain regional identity of our iPSC-derived neuronal cells. While transcriptome profiles from NPC best reflects in vivo neuronal tissue from fetal brains at 4–10 post-conception weeks (pcw) and are most likely to be of cortical identity, the neurons, after 4 weeks of differentiation from NPC, best reflect a mid-fetal period (16–24 pcw) and have an expression profile more similar to cerebellar cortex, evidencing a temporal progression of neuronal differentiation (Fig. 1e, f). Importantly, the mid-fetal temporal window has already been shown to be a critical period for ASD pathophysiology [45]. Finally, using panels of specific cell-type markers, we found that our neuronal culture is characterized by the presence of GABAergic and glutamatergic neurons, with no evidence for the presence of oligodendrocytes and dopaminergic or serotoninergic neurons (Fig. 1g; Supplementary Table S5). All these results validate our differentiation protocol and transcriptional data, particularly revealing that the neurons derived using this protocol have a transcriptional profile more similar to fetal brains of a specific developmental window that is relevant for ASD risk.

### Transcriptome analysis reveals altered gene expression in ASD that correlates with neuronal morphological abnormalities

**Fig. 2:**
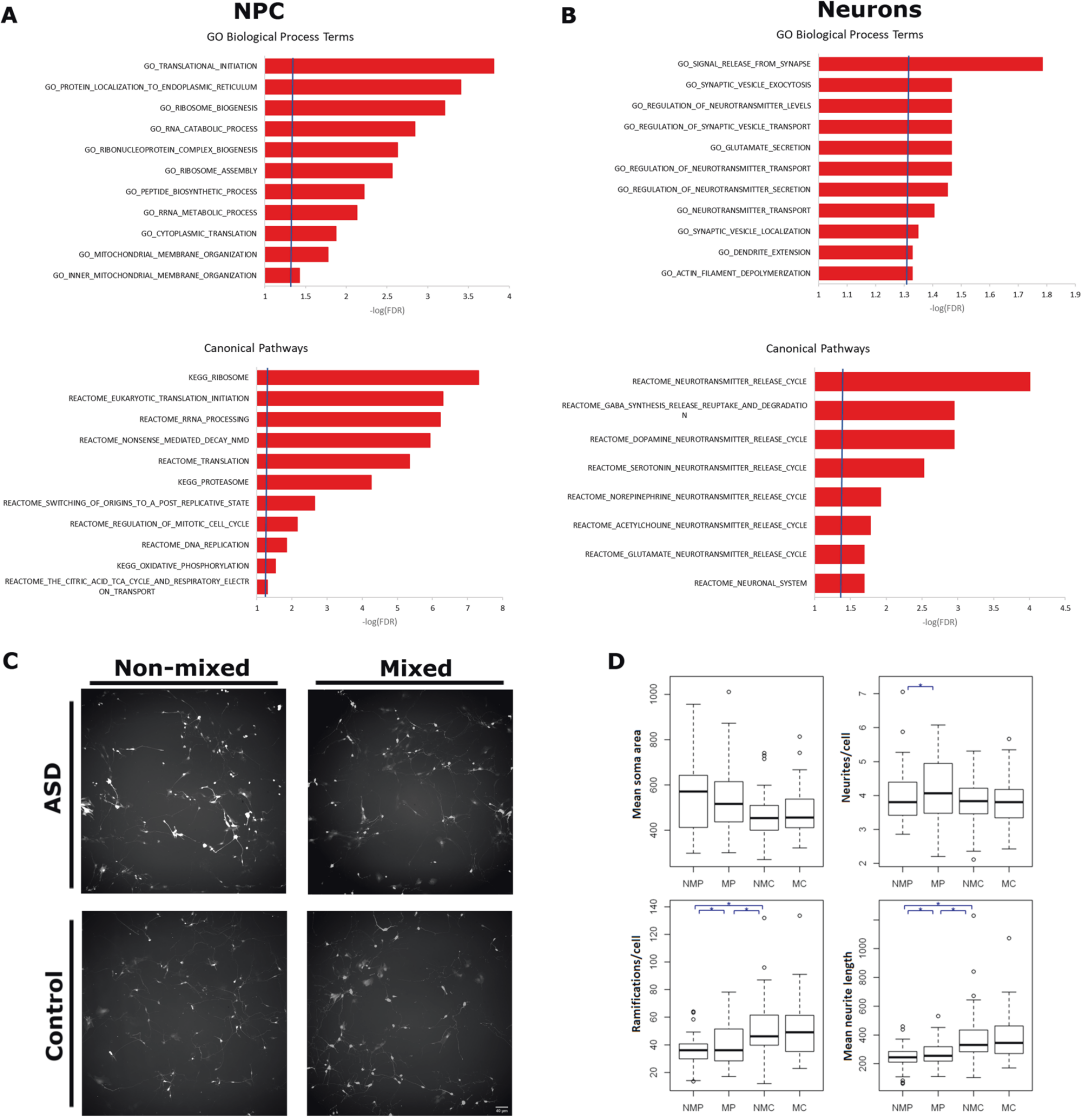
Functional annotation analysis of differentially expressed genes in NPC and neurons from ASD individuals. **a** Functional annotation analysis shows that top upregulated genes in ASD NPC are enriched (red) within Gene Ontology (GO) terms and canonical pathways related to ribosome biogenesis, translation regulation and mitochondrion function. **b** Top ranked upregulated genes in ASD neurons (red) are enriched within GO terms and pathways related to synaptic signaling, neurotransmitter release and dendrite extension. A full description of the functional annotation analysis is available in Supplementary Tables 6 and 7. **c** Illustrative images of ASD neurons co-cultured with non-GFP neurons from the same individual or non-GFP neurons from a control, and control neurons co-culture with non-GFP neurons from the same individual or from an ASD individual. **d** Box plots showing the quantification of soma size, neurites per cell, number of ramifications per cell and neurite length for all these conditions. NMP: non-mixed patient (*n* = 3 clones); MP: mixed patient (*n* = 3 clones); NMC: non-mixed control (*n* = 5 clones); MC: mixed control (*n* = 5 clones). At least eight photos per individual per condition were analyzed.

Since we have multiple clones for each individual, we then applied a statistical model that accounts for a repeated measurement design [26] to properly control the false discovery rate in the identification of differentially expressed genes (DEGs) between ASD and control neuronal cells. In NPC, we did not identify any gene with differential expression that reached statistical significance (Supplementary Table S6), while in neurons, we found 20 genes with significant expression dysregulation (multiple testing corrected *p* < 0.05, Supplementary Table S7). Remarkably, three of these genes (*KCNB1, SLC12A5, CHMP1A*) are associated with ASD according to Sfari database. These low numbers of statistically significant DEGs are not unexpected in a heterogeneous disease such ASD, as not necessarily all patients will have expression dysregulation of the exact same genes. Based on that, we applied a functional enrichment method for our data set not focused on specific set of genes defined by an arbitrary *p*-value, but instead, that evaluates if a set of genes involved in a particular function is highly ranked relative to other genes in terms of differential expression [28]. We found that, in NPC, the highly ranked upregulated genes are enriched within functions related to translational process, mitochondrion organization and function and cell cycling (Fig. 2a, Supplementary Table S6). On the other hand, in neurons, synapse, neurotransmitter release, as well as actin filament and dendrite extension regulation are the biological processes and pathways found as enriched for highly ranked upregulated genes (Fig. 2b, Supplementary Table S7). The highly ranked downregulated genes in both NPC and neurons were not found as enriched within any biological process or canonical pathway (Supplementary Tables S6 and S7).

In order to functionally validate these expression differences, we analyzed morphological phenotypes in ASD and control neurons. For that, after four weeks of differentiation from NPCs, we sorted the GFP-expressing neurons, re-plated them in low density with non-GFP-expressing population of differentiated neuronal cells either from the same individual (non-mixed condition—NM) or from an individual from the opposed group (mixed condition—M), and cultured the cells for 4 more days to have extended neurites again (Fig. 2c, d). In the non-mixed condition, we found that ASD neurons showed the same number of neurites per cell as compared with controls, but these neurites displayed ~68% less ramifications (*p* = 0.008) and were ~65% shorter (*p* = 0.005; Fig. 2c, d; Supplementary Table 8). Interestingly, when the neurons of the patients were co-cultured with control-derived populations of neuronal cells, there was an improvement of neurite morphology: in comparison to the non-mixed condition, the number of ramifications and the length of the neurites of ASD neurons increased ~22% (*p* = 0.016 and *p* < 0.001, respectively), which, however, is still slightly different from control neurons (*p* < 0.05; Fig. 2c, d; Supplementary Table 8). Although we found no differences in the number of neurites per ASD-derived neuron compared with controls in the non-mixed condition, this number was increased when the neurons were co-cultured with control population (*p* < 0.001; Fig. 2d; Supplementary Table 8). On the other hand, no difference was noted in these parameters when control neurons were co-cultured with ASD population of neuronal cells compared with the non-mixed condition (Fig. 2d; Supplementary Table 8). These morphological abnormalities functionally corroborate the expression changes found in genes related to dendrite extension and actin cytoskeleton dynamics in ASD neurons. Moreover, the slightly improvement achieved in such morphological parameters once ASD neurons were co-cultivated with control neurons suggests an impairment in neuronal signaling, another function found as enriched for highly ranked upregulated genes in our transcriptome analysis (Fig. 2b, Supplementary Table S7).

### Networks of co-expressed genes implicated in translational processes and neurotransmission are associated with ASD

As previously mentioned, in a heterogeneous disease such as ASD, it is not expected that different patients will have dysregulation of the same set of genes, but instead, they might present dysregulation of different genes that participates in a same functional network. Therefore, in order to explore the data in a system level context, we used weighted-gene co-expression network analysis (WGCNA) [27] to identify groups of co-regulated genes in NPC and neurons. We also conducted a module-preservation analysis [29] in order to validate the biological nature of the detected modules and evaluate whether NPC and neurons co-expression networks were consistently well preserved in fetal brain cortices at 4–10 pcw and at 16–24 pcw, which are, respectively, the fetal periods best correlated with our iPSC-derived neuronal cell transcriptome profiles.

**Fig. 3:**
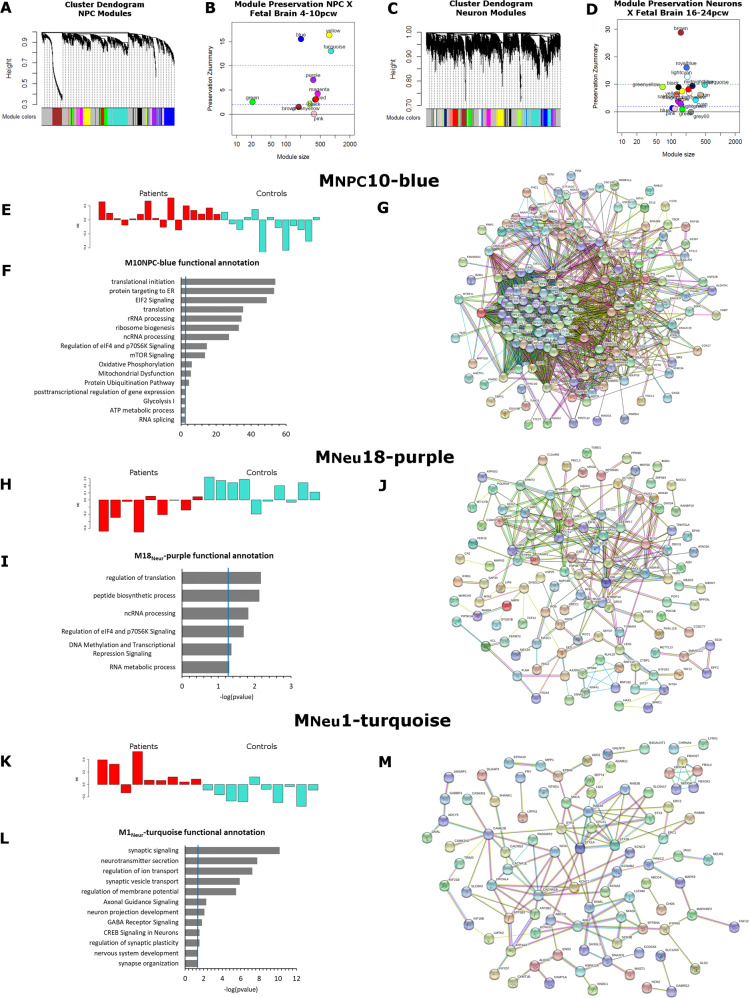
Modules of co-expressed genes in NPC and neurons are dysregulated in ASD. **a**–**d** Network analysis dendrogram showing clustering of genes based on topological overlap for identification of modules of co-regulated genes in iPSC-derived NPC (**a**) and neurons (**c**) and *z*-scores of preservation of such modules in transcriptome data from fetal brain samples (**b** for NPC modules and **d** for neuron modules). **e**, **h**, **k** Module eigengenes for M_NPC_10-blue, M_Neu_18-purple, and M_Neu_1-turquoise showing, respectively, upregulation, downregulation, and upregulation of these modules in ASD (red) compared with control (turquoise) samples. **f**, **i**, **l** Functional annotation enrichment analysis for the three ASD-associated modules. **g**, **j**, **m** Protein–protein interaction networks showing biological evidence of interaction of the 200-top genes assigned to each of the co-expression networks identified by WGCNA. Not connected nodes were hidden.

In NPC, we identified 11 modules of co-expressed genes (Fig. 3a; Supplementary Table S9), most of which are moderately (2 < *Z*_score_ < 10) or strongly (*Z*_score_ < 10) preserved in transcriptome data from fetal brain cortices at 4–10 pcw (Fig. 3b). One of these modules, M_NPC_10-blue, is significantly correlated with disease status (*P*_bonferroni_ = 0.044; Supplementary Table S9), presenting as upregulated in ASD individuals (Fig. 3e). M_NPC_10-blue is enriched for genes related to protein biosynthesis, RNA processing and splicing, and mitochondrial function (Fig. 3f).

Twenty modules of co-expressed genes were identified in neurons, which showed moderate to strong evidence of preservation in fetal brain cortices at 16–24 pcw (Fig. 3c, d). Two of these modules, M_Neu_1-turquoise and M_Neu_18-purple, were found to be significantly correlated with the disease status (Supplementary Table S10). They presented a significant inverse correlation (cor = −0.79, *p* = 4 × 10^−5^), showing that they have an inverse co-expression relationship. M_Neu_18-purple (*P*_bonferroni_ = 0.048) is downregulated in ASD patients (Fig. 3h) and is enriched for genes related to translational processes and regulation of gene expression (Fig. 3i). M_Neu_1-turquoise (*P*_bonferroni_ = 0.02), on the other hand, is upregulated in ASD individuals (Fig. 3k). Accordingly, its functional annotation showed similar results to the gene-by-gene differential expression analysis: M_Neu_1-turquoise is enriched for genes related to synapse, ion channels, neurotransmission, and genes involved in signaling pathways important to neuronal function, such as GABA receptor signaling and axonal guidance signaling (Fig. 3l; Supplementary Table S10).

Importantly, the biological nature of these three ASD-associated modules is supported by protein–protein interaction evidence (Fig. 3g, j, m) and by strong to moderate evidence of module preservation in fetal brain cortices (M_NPC_10-blue: *Z*-score = 16; M_Neu_18-purple: *Z*-score = 3; M_Neu_1-turquoise: *Z*-score = 9.9; Fig. 3b, d).

### Upregulation of M_NPC_10-blue in NPC may trigger expression alteration of ASD-associated modules in neurons during neuronal differentiation by changes in translational level

**Fig. 4:**
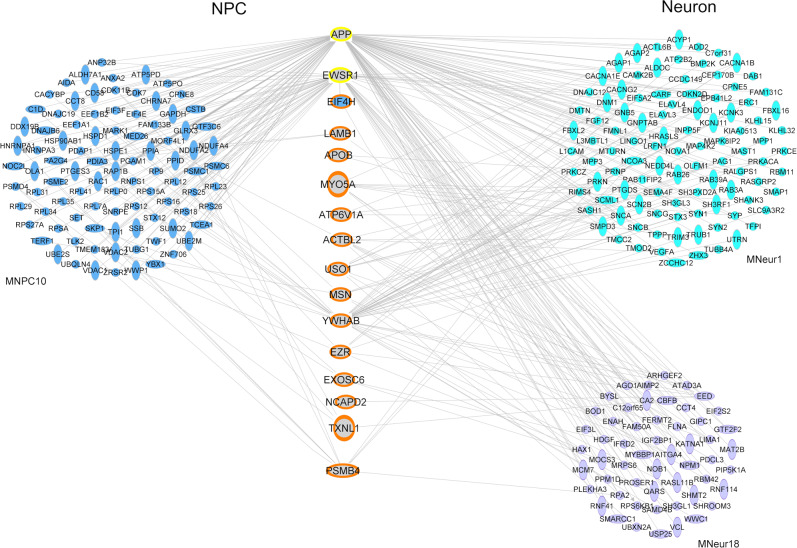
Putative molecular links between ASD dysregulated modules in NPC and neurons. Differentially regulated proteins in NPC that act as upstream regulators of a significant number of genes from M_Neu_1-turquoise (yellow outline) and that have expression levels significantly correlated with M_NPC_10-blue and M_Neu_1-turquoise/M_Neu_18-purple eigengenes (orange outline) are shown in the center of the figure. EIF4H, which is dysregulated in transcription and protein levels, is marked in blue as this molecule is also a member of M_NPC_10-blue. These proteins are connected with genes from the three ASD-associated modules which they are known to interact with.

We then hypothesized that the overexpression of M_NPC_10-blue in ASD NPC could trigger the dysregulation of key molecules from MNeu1-turquoise or MNeu18-purple early in neuronal differentiation (before the establishment of these co-expression modules) and thus, as the differentiation process proceeds, the altered expression of these genes would lead to the dysregulation of the entire modules, as they start to emerge in patients’ neurons. To explore this possible relationship, we first addressed whether M_NPC_10-blue genes would act as upstream regulators of M_Neu_1-turquoise or M_Neu_18-purple, but we did not find any M_NPC_10-blue gene whose downstream targets are significantly enriched for genes belonging to the two neuron modules (Supplementary Table S11). Considering that M_NPC_10-blue is mainly composed of genes involved in translational processes, we hypothesized that the dysregulation of this module at the transcription level could be leading to an aberrant expression of specific proteins in NPC which, in turn, might control the expression of M_Neu_1-turquoise or M_Neu_18-purple genes. Thus, we conducted a proteomic study in NPC of 3 ASD patients and 3 control individuals and found that two differentially expressed proteins in ASD NPC, EWSR1 (*P*_anova_ = 0.02, fold change = 1.48) and APP (*P*_anova_ = 0.04, fold change = 1.5), are upstream regulators of a significant number of M_Neu_1-turquoise genes (Fig. 4; *p* < 0.05, see Supplementary Table S11).

Next, to further explore the possible regulatory links between these modules, we verified the correlation between the NPC protein expression levels and M_NPC_10-blue, M_Neu_1-turquoise and M_Neu_18-purple module eigengenes of the same samples included in the proteomic analysis. We found 14 proteins whose expression levels are significantly correlated to both M_NPC_10-blue and M_Neu_1-turquoise module eigengenes and 6 proteins with significant correlation to M_NPC_10-blue and M_Neu_18-purple module eigengenes (Fig. 4, Supplementary Table S11). The expression levels of two of these proteins are significantly correlated to module eigengenes of the three modules: EIF4H, which is upregulated in ASD NPC (*P*_anova_ = 0.003, fold change = 3.5), and LAMB1, which is downregulated in ASD NPC (*P*_anova_ = 0.02, fold change = 1.5). Remarkably, *EIF4H* is also a member of M_NPC_10-blue and interacts with the M_Neu_1-turquoise upstream regulator EWSR1 (Fig. 4; Supplementary Table 11). Taken together, these exploratory analyses point to some putative proteins that may act as molecular links between NPC and neuron transcriptome dysregulation seen in ASD patients.

### Genetic evidence supports the association of M_Neu_1-turquoise with ASD

**Fig. 5:**
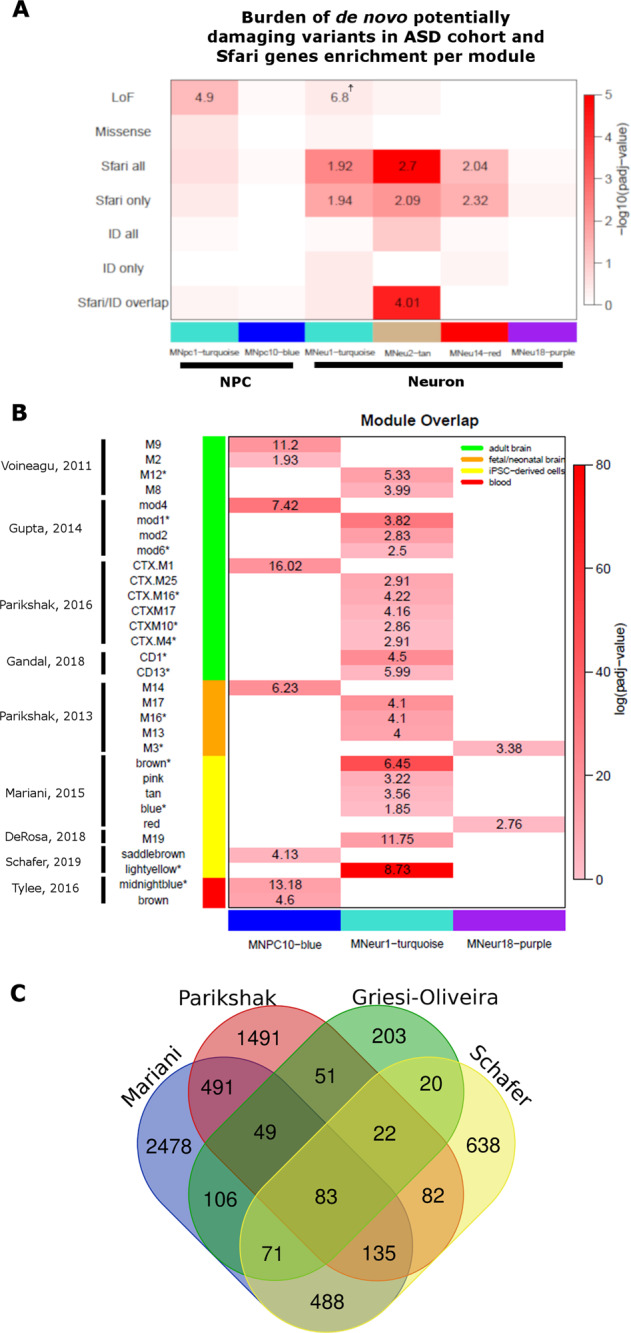
Consistent association of MNeu1-turquoise with ASD. **a** Genetic evidence for M_Neu_1-turquoise involvement in ASD pathophysiology: the top part of the panel shows the burden of de novo potentially damaging LoF and missense variants in ASD cohort for the indicated modules, while the bottom part shows enrichment for Sfari and/or ID related genes. Heatmap colors refers to FDR values of enrichment trend. Odds-ratio values are only plotted for those modules that showed enrichment for genes in the denoted category (FDR < 0.05; OR > 1), except for the burden of ASD patients harboring LoF variants in M_Neu_1-turquoise (☨) which, although not statistically significant, presented a clear trend of enrichment. FDR and OR values for the full set of modules are available in Supplementary Table S12. **b** Module overlap analysis comparing the three ASD-associated modules identified in this study with a series of transcriptome studies conducted either with postmortem brain samples or iPSC-derived neuronal cells (as indicated by the legend at left). All the modules that presented significant overlap with M_NPC_10-blue, M_Neu_1-turquoise, or M_Neu_18-purple (FDR < 0.05, OR > 1) are shown and modules associated with ASD in each of the selected studies are indicated by an asterisk. Modules correspondent to M_NPC_10-blue were identified in all the selected studies and, although not associated with ASD in none of the studies conducted with neuronal samples, it was detected as dysregulated in ASD patients in a metanalysis investigation of blood transcriptome. A M_Neu_18-purple correspondent module was identified in fetal brain samples and such module was found as enriched for LoF damaging variants present in ASD patients. M_Neu_1-turquoise correspondent modules were identified in all the studies and, in all of them, they were found as dysregulated in ASD patients. Heatmap colors refers to FDR values of enrichment trend and odds-ratio values are plotted (FDR < 0.05; OR > 1). For detailed overlap analysis, refers to Supplementary Table S13. **c** Venn-diagram showing the number of genes in overlap between the different M_Neu_1-turquoise correspondent modules identified in studies conducted with either fetal neuronal brain or iPSC-derived neurons.

Taking advantage of the large exome-sequencing datasets of ASD families already published [33, 34], we examined whether individuals with ASD (*n* = 3982) had an increased burden of de novo LoF or rare missense variants compared with their siblings (*n* = 2078) in any of the modules identified either in NPC or neurons, particularly those that were found as ASD-associated. We only considered LoF variants in LoF-intolerant genes (pLi ≥ 0.8) and missense variants predicted to be potentially damaging by CADD-score (CADD ≥ 20). We observed that ASD patients show a marginally increased burden of LoF variants in M_Neu_1-turquoise genes (*p* = 0.044, OR = 6.8, Supplementary Table S12), which does not exceed the statistical significance after FDR correction (*P*_adj_ = 0.36, Fig. 5a). It is interesting to note that among the 2078 control subjects in this cohort, only one female individual shows a LoF variant in a M_Neu_1-turquoise gene (*JAKMIP1*), which is also shared with her male ASD sibling, while 13 out of 3982 ASD patients harbor LoF variants in M_Neu_1-turquoise genes (Supplementary Table S12), showing that, despite the lack of significance, a much higher proportion of ASD patients presents LoF variants in genes belonging to this module than control individuals. In addition, we unexpectedly found an increased burden of LoF variants in ASD patients in genes from a module associated with chromatin and transcription regulation identified in NPC (Supplementary Table S9), module M_NPC_1-turquoise, which did not present expression dysregulation in our patients (Fig. 5a, Supplementary Table S12).

In our cohort in particular, although we did not identify any rare de novo disrupting variant of strong effect in M_NPC_10-blue, M_Neu_1-turquoise, or M_Neu_18-purple genes in any of the six patients, we identified some inherited rare potentially damaging variants in genes of these modules, which are described in Supplementary Table S4.

We next asked whether any of the modules of co-expressed genes identified in NPC and neurons are enriched for ASD-risk genes compiled by the Sfari database (https://gene.sfari.org/). We observed an enrichment of Sfari genes (“Sfari all”, Fig. 5a) in three neuron modules: M_Neu_1-turquoise (FDR = 0.004, OR = 1.94), M_Neu_2-tan (FDR = 8.9 × 10^−7^, OR = 2.7), and M_Neu_14-red (FDR = 0.04, OR = 2.04). Because intellectual disability (ID) is frequently found as a comorbidity among patients with ASD and, in fact, many genes are associated with both conditions, we next considered only Sfari genes that are not associated with ID to address the modules specificity to ASD. This analysis revealed an enrichment for ASD-exclusive genes (“Sfari only”) again in the same three modules, whereas no enrichment was found for “ID all” or “ID only” genes in any module (Fig. 5a, Supplementary Table S12). Remarkably, M_Neu_2-tan is the only module enriched for genes associated with both ASD and ID (Fig. 5a, “Sfari/ID overlap”, FDR = 4.3 × 10^−5^, OR = 4) and it is slightly more enriched for “Sfari all” genes (OR = 2.7) than for “Sfari only” genes (OR = 2.09), suggesting a possible association of this module with comorbid ID. Collectively, the high frequency of LoF mutations and the enrichment for Sfari genes in M_Neu_1-turquoise bring support to the association of this module to ASD.

### The synapse-related module M_Neu_1-turquoise has been consistently associated with ASD in several independent gene expression studies using neuronal cells

In order to validate our findings, we next asked whether the ASD-associated modules identified in this study were also found as disease-associated in other expression studies conducted with ASD neuronal cells either obtained from postmortem brains [8–10, 39] or derived from iPSC [16, 17, 19]. We also compared our results to the modules identified in fetal/neonatal brain samples from BrainSpan database that are enriched for ASD candidate genes [38] and to modules identified in blood samples from ASD patients [37], to verify whether the dysregulation of any of the ASD-associated modules could be detected in an easily accessible tissue (Fig. 5b; Supplementary Table S13).

Although M_NPC_10-blue showed a significant overlap with modules identified by other studies using postmortem brains and iPSC-derived neurons (Fig. 5b, Supplementary Table S13), these M_NPC_10-blue correspondent modules were not found as ASD-associated in any of such studies (ASD-associated modules are denoted by an asterisk in Fig. 5b). Concordantly, we identified a module in iPSC-derived neurons, M_Neu_19-greenyellow, that also has a significant overlap with M_NPC_10-blue (*p* = 7.3 × 10^−13^; OR = 13.11; data not shown) but it is not dysregulated in our cohort of patients compared with controls. On the other hand, M_NPC_10-blue shows a significant overlap with a module identified as upregulated in blood of autistic patients (mod midnightblue, *p* = 8.3 × 10^−14^, OR = 13.18).

M_Neu_18-purple has a moderate overlap with module red from Mariani et al. [16] (*p* = 0.001, OR = 2.76), which is not associated with ASD, and with module M3 from Parikshak et al. [38] (*p* = 3.3 × 10^−5^, OR = 3.37) which, in turn, is enriched for de novo variants found in ASD individuals (Fig. 5b).

M_Neu_1-turquoise also has a significant overlap with a module enriched for de novo protein-disrupting variants seen in ASD [38], the module M16 (*p* = 7.7 × 10^−14^, OR = 4.09, Fig. 5b). Most importantly, M_Neu_1-turquoise has a very strong overlap with modules identified as dysregulated in ASD in all selected expression studies using neuronal cells (Fig. 5b): M12 from Voineagu et al. [8] (*p* = 2.8 × 10^−14^, OR = 5.33), mod1 and mod6 from Gupta et al. [9] (*p* = 8.2 × 10^−31^, OR = 3.82 and *p* = 7.5 × 10^−5^, OR = 2.49, respectively), CTX16, CTX10, and CTX4 from Parikshak et al. [10] (*p* = 2.5 × 10^−9^, OR = 4.22; *p* = 0.01; OR = 2.85; *p* = 0.01, OR = 2.91, respectively), CD1 and CD13 from Gandal et al. (*p* = 3.3 × 10^−22^, OR = 4.5 and *p* = 8.5 × 10^−5^, OR = 5.99, respectively), modules brown and blue from Mariani et al. [16] (*p* = 1.1 × 10^−48^, OR = 6.45 and *p* = 0.01, OR = 1.84, respectively), M19^d35^ from De Rosa et al. [19] (*p* = 1.7 × 10^−15^, OR = 11.74) and module lightyellow (TM1) from Schafer et al. [17] (*p* = 10^−80^, OR = 8.73). Curiously, while the modules brown and blue from Mariani’s study, M19^d35^ from De Rosa’s study and module lightyellow (TM1) from Schafer’s study, which were all also conducted in iPSC-derived neurons, are upregulated in ASD individuals such as the M_Neu_1-turquoise in the present study, the ASD-associated modules identified in postmortem brain tissue are downregulated in the patients. These results show the reliability of M_Neu_1-turquoise as an ASD-associated module of co-expressed genes, since it has been consistently detected as differentially expressed in a series of studies that either used ASD postmortem brain samples or iPSC-derived neuronal cells.

Finally, we sought to further refine the list of genes within M_Neu_1-turquoise, capturing only those genes with a strong evidence of being part of this module. For this purpose, we identified which genes were consistently assigned to any of the modules with significant overlap with M_Neu_1-turquoise in all the studies that used either fetal/neonatal brain samples or iPSC-derived neurons [16, 17, 38] (Fig. 5c; see the Materials and methods section). Remarkably, this approach seems to prioritize ASD-risk genes, since the list of 83 genes found in this overlap is more enriched for Sfari genes than the lists of the original modules (Fig. 5c; Table 1; Supplementary Table S13), suggesting that this set of genes should be carefully examined in exome sequencing from ASD patients.

**Table 1:** Enrichment for Sfari genes in each of the M_Neu_1-turquoise correspondent modules identified in the denoted studies.

Study	Module	Sfari genes	Total genes in module	% of Sfari genes	Odds-ratio	*P*_adj_
Griesi-Oliveira	MNeu1	44	506	8.7%	2.07	0.0001
Mariani et al. [16]	Brown	113	1033	10.9%	3.07	3 × 10^−19^
	Blue	80	1354	5.9%	1.44	0.007
	pink	37	654	5.7%	1.34	0.14
	tan	19	404	4.7%	1.09	0.81
Parikshak et al. [38]	M13	37	699	5.3%	1.28	0.21
	M16	45	455	9.9%	2.59	1 × 10^−6^
	M17	57	810	7.0%	1.78	0.0004
Schafer et al. [17]	Lightyellow	49	1305	3.8%	1.03	0.81
Consensus module		12	83	14.5%	3.33	0.001

## Discussion

Analysis of differential transcriptional profile can be useful to detect more homogeneous biological alterations among ASD individuals, helping to uncover pathways consistently compromised in patients [46, 47], to interpret which genomic variants might have stronger functional effects and, ultimately, may allow the identification of diagnostic biomarkers. Because ASD is an extremely heterogeneous disease, the investigation of different subgroups of patients regarding genetic underlying causes, functioning level and comorbidities will be important to understand whether they share common transcriptome alterations or whether particular expression differences can be attributed to each of these groups.

Here, we analyzed the transcriptome of iPSC-derived NPC and neurons of controls and an ASD sample enriched with high-functioning normocephalic patients in whom the disease probably follows an oligogenic or polygenic pattern of inheritance, as we did not identify any rare de novo potentially damaging loss-of-function mutation that could account in isolation for the disease in these individuals. This is in agreement with previous data showing that de novo damaging variants are more associated with lower IQ [32, 48], giving further support to the hypothesis that high-functioning patients most probably follow complex pattern of inheritance, where medium to low risk inherited variants may play a major role.

We validated our transcriptional data demonstrating that they are comparable to transcriptional profiles of human fetal brains, and the transition from iPSC-derived NPC to neurons indeed mimics the temporal progression of in vivo brain development, since the gene expression signature of NPC correlates with an earlier period of gestation compared with neurons. Particularly, our 4 weeks long differentiation protocol generated neurons showing a transcriptional signature whose the highest correlation is with a temporal window of development that seems to be critical for ASD pathophysiology, since there is an enrichment of ASD-associated genes among genes expressed during this period, as previously proposed [45]. Our study reinforces the conclusion made by these authors, since we demonstrate that, indeed, there are important differences in gene expression of ASD individuals in this time point. The correlation with mid-early fetal brain expression profile has already been shown by another study using ASD iPSC-derived neurons [16], which was conducted using 3D neuronal organoids. Thus, our results suggest that iPSC-derived neurons at the fourth week of differentiation generated on 2D is a suitable model to explore transcriptional dysregulation in ASD or other neurological conditions whose the critical time period for disease pathophysiology is the mid-early gestational period.

To identify genes with differential expression, we used a statistical method that accounts for the repeated measures of different clones of a same individual, which is an important aspect of the design of the experiments in iPSC field that is often neglected. Although few genes reached statistical significance for differential expression in ASD neuronal cells, which is in agreement with the complexity of the disease, the top ranked upregulated genes showed enrichment within functions and pathways relevant for ASD.

Our systems biology approach revealed expression dysregulation in NPC from ASD individuals, showing that there might be molecular alterations in earlier neurodevelopmental phases in these patients as well. Whereas the co-expression module dysregulated in ASD NPC, M_NPC_10-blue, is not enriched for Sfari genes and the frequency of ASD individuals harboring disrupting variants in genes of this module is not different from controls, thus lacking genomic evidence of its involvement in ASD pathophysiology, a similar module was identified as dysregulated in a meta-analysis of transcriptome studies conducted in the blood of autistic patients [37]. Thus, the expression levels of core genes within this network should be further explored in blood, in order to verify if they might be potentially used as a biomarker for the disease. Curiously, although M_NPC_10-blue is preserved in neurons according to our module overlap analysis, it is not dysregulated in this type of cells in ASD individuals, suggesting that its expression levels are normalized in the patients once NPC differentiate to neurons.

M_NPC_10-blue is enriched for genes involved in protein synthesis, a cellular process already implicated in ASD pathophysiology (reviewed in Bourgeron [6]). We hypothesized that its expression alteration could dysregulate the levels of particular proteins which, during neuronal differentiation process, would control expression of hub genes that are important for the establishment of M_Neu_1-turquoise and M_Neu_18-purple co-expression modules, the two networks found as ASD-associated in neurons. Indeed, using proteomic analysis, we identified some potential molecular links between these three modules, such as EWSR1, APP and EIF4H. Particularly, *EIF4H* is one of the genes deleted within 7q11.23 in patients with Williams Syndrome, which shares some phenotypic characteristics seen in ASD [49], while altered levels of APP and its metabolites have been identified in brain and plasma from autistic patients [50]. Although these results should be interpreted with cautious due to the limited number of samples included in this analysis, these possible molecular regulatory links between modules in NPC and in neurons deserve further investigation.

Alternatively, the upregulation of M_Neu_1-turquoise, a module of synapse genes, and downregulation of M_Neu_18-purple, which is enriched for translation related genes, could be induced primarily by genetic variants in genes belonging to these modules. Although we have not found a statistically significant excess of ASD individuals with disrupting variants in genes from M_Neu_1-turquoise and M_Neu_18-purple compared with controls, we did observe a clear trend toward this enrichment in M_Neu_1-turquoise. Also, other studies have found enrichment of genes harboring de novo LoF variants identified in ASD individuals in modules that overlap M_Neu_1-turquoise and M_Neu_18-purple (M16 and M3, respectively, in Parikshak et al. [38] and replicated by Takata et al. [51]). On the other hand, considering that our transcriptome analysis was done in non-monogenic ASD cases (as it is the case of other studies such as Mariani et al. [16] and Schafer et al. [17]), it is likely that the de novo variants are actually not a major driving factor for expression alteration of these genes, and rare and/or common inherited variants might also play a role in the regulation of the modules. Accordingly, different studies have shown that M_Neu_1-turquoise correspondent modules are enriched for ASD positive GWAS signals [8–10, 39]. In addition, Schafer and colleagues [17] have recently demonstrated that ASD NPC present altered chromatin accessibility in close distal regions of genes from the M_Neu_1-turquoise correspondent module identified in their study (lightyellow-TM1), which could be explained by variants in such regulatory elements. The distribution of rare inherited variants across these modules, particularly in regulatory regions, should be further explored in large genome sequencing studies that are being conducted in order to provide a better comprehension of the primary mechanisms driving their expression alterations.

We also found an enrichment of Sfari genes in M_Neu_1-turquoise, reinforcing the body of evidence to support the association of this module with ASD. In addition, M_Neu_1-turquoise is enriched for genes related to synaptic transmission, voltage-gated channels function, dendrite extension and neuronal signaling. Our neuronal morphological analysis showed that ASD individuals have less arborized neurons with shorter neurites, and these phenotypic alterations slightly improve when ASD neurons are co-cultivated with control neurons. Similar morphological alterations were already found in other syndromic and non-syndromic ASD individuals [11, 14, 52]. Other studies have also reported altered excitability and synaptic function in idiopathic ASD iPSC-derived neurons [15, 53, 54]. These results give functional support for the role of M_Neu_1-turquoise expression dysregulation in ASD pathophysiology.

Moreover, comparing our results with multiple transcriptome studies of neuronal cells, we found that M_Neu_1-turquoise has a strong overlap with ASD-associated modules identified by all these studies, revealing the consistent association of this co-expression network of synapse genes with ASD. Interestingly, this module has been consistently found as upregulated in iPSC-derived neurons [16, 17, 19] and downregulated in postmortem brain tissue [8–10, 39]. Some hypotheses can be formulated to try to explain this apparent discrepancy: first, postmortem brain tissue is a mixture of different cell types, including neurons and glial cells. If the brains of ASD individuals have fewer neurons than controls, an upregulation of the module can be masked by the reduced number of neurons, inducing a misleading interpretation of a downregulation of the module. The use of a normalization method accounting for the proportion of neurons, as applied here, to analyze postmortem brain data would help to address this hypothesis. On the other hand, it has been shown that M_Neu_1-turquoise correspondent modules (represented by M13, M16, and M17 in Parikshak et al. [38] and M15 in Kang et al. [24]) present a gradual increase in expression from the early fetal period until around 1–2 years old, when the module expression reaches a plateau. In parallel, a recent study identified heterochronic dynamics in the regulation of this module of synaptic genes, which initiates the increase in expression much earlier in ASD individuals than in controls [17]. It might be possible, then, that an overexpression of M_Neu_1-turquoise at an improper period of neurodevelopment would induce a negative feedback of regulation, repressing these genes later on, or refraining their continuous increased expression, leading to the downregulation of the module postnatally.

Importantly, this module has been identified as dysregulated in iPSC-derived neurons regardless the endophenotype that was studied (macrocephalic patients in Schafer et al. [17] and Mariani et al. [16], low-functioning patients in DeRosa et al. [19], and high-functioning patients in the present study), suggesting that this network is a core module for ASD pathophysiology. Whether other discrete transcriptional alterations can distinguish different endophenotypes is a question that remains to be explored. In this regard, it will be important that different studies use standardized methods and provide a detailed clinical and genetic characterization of the sample, in order to allow a reliable comparison across the studies. We found some interesting results that may guide future efforts attempting to address this question. For instance, we found genetic evidence supporting the association of M_NPC_1-turquoise with ASD, a module not dysregulated in patients’ NPC in our expression analysis, which is enriched for genes related to chromatin regulation. Interestingly, abnormal chromatin accessibility was recently identified in NPC from ASD macrocephalic individuals [17]; therefore, it would be worthy to investigate if this module is particularly dysregulated in this subset of patients. In addition, a module that is not dysregulated in ASD neurons in our sample, M_Neu_2-tan, was found as particularly enriched for Sfari genes that are also related to ID, differently than M_Neu_1-turquoise. These results, along with the fact that our sample is enriched with high-functioning patients, suggest that dysregulation of M_Neu_2-tan might contribute to ID as a comorbidity in ASD, and deserve further investigation in samples enriched with low-functional individuals. Finally, it is interesting to notice that modules of co-expressed genes related to immune response have been consistently found as dysregulated in ASD postmortem brain tissue [8–10, 39], while in transcriptome studies with iPSC-derived neurons, they have not. Since this module is associated with glial markers, this result may be due to a low proportion of glial cells in the samples derived from iPSC (and particularly, in our case, this could have been even more minimized due to the normalization step based on the proportion of neurons that we have conducted). Future complementary transcriptome analyses on iPSC-derived glial cells are worthy to explore whether the in vitro model will also reflect the dysregulation of this immune response module that is seen in postmortem brains, as it is the case for the module of synapse genes. Another unexplored question worthy to be pursued is whether differential splicing events can also be detected in iPSC-derived neuronal cells, possibly reflecting what happens during a fetal period, as it has been seen in transcriptome analysis of ASD postmortem brains [55].

Although the small sample size imposes some limits to this study, taken together, our results support the idea that ASD patients are very homogeneous relatively to the expression profile of the module of synapse genes, M_Neu_1-turquoise, since it has been identified as dysregulated in a series of transcriptome studies, regardless the endophenotypic group analyzed. The dysregulation of this module of co-expressed synaptic genes can be considered one of the most consistent findings to be demonstrated to date for ASD, suggesting its central role for the disease pathophysiology. Therefore, this module should be carefully considered when exploring genetic variants in ASD individuals, its expression pattern might be used as a biomarker for the disease and, specially, it should be explored as a potential target for disease treatment.

## Supplementary information

Supplementary Methods

Supp Fig. S1

Supp Fig. S2

Supp Fig. S3

Supp Table S1

Supp Table S2

Supp Table S3

Supp Table S4

Supp Table S5

Supp Table S6

Supp Table S7

Supp Table S8

Supp Table S9

Supp Table S10

Supp Table S11

Supp Table S12

Supp Table S13

Supplementary Figure Legends
