# Supplementary material for: Transcriptome of iPSC-derived neuronal cells reveals a module of co-expressed genes consistently associated with autism spectrum disorder
Source: Mol Psychiatry. 2020 Feb 14;26(5):1589–605. doi: 10.1038/s41380-020-0669-9 (PMC8159745; doi:10.1038/s41380-020-0669-9)
Supplement: Supplementary file 1 — Supplementary Methods [file 41380_2020_669_MOESM1_ESM.docx]

**Supplementary Methods**

**Transcriptome of iPSC-derived neuronal cells reveals a module of co-expressed genes consistently associated with autism spectrum disorder**

Griesi-Oliveira, K., PhD^1,2*^; Fogo, M.S., MSc^1,2^; Pinto, B.G.G.., BSc^1^; Alves, A.Y., BSc^1^; Suzuki, A.M., MSc^2^; Morales, A.G., PhD^2^; Ezquina, S., PhD^2^; Sosa, J.O., PhD^3^; Sutton, G.J., BSc^4^; Sunaga-Franze, D.Y., PhD^5^; Bueno, A.P., MD^1^; Seabra, G., BSc^6,7^; Sardinha, L., PhD^1^; Costa, S.S., MSc^2^; Rosenberg, C., PhD^2^; Zachi, E.C., PhD^8^; Sertie, A.L., PhD^1^; Martins-Souza, D., PhD^6,7^; Reis, E.M., PhD^4^; Voineagu, I., PhD^5^; Passos-Bueno, M.R., PhD^2*^.

1. Hospital Israelita Albert Einstein, São Paulo, Brazil, 2. Departamento de Genética e Biologia Evolutiva, Instituto de Biociências, Universidade de São Paulo, São Paulo, Brazil, 3. Departamento de Bioquímica, Instituto de Química, Universidade de São Paulo, São Paulo, Brazil, 4. School of Biotechnology and Biomolecular Sciences, University of New South Wales, Sydney, Australia, 5. Max Delbrück Center for Molecular Medicine in the Helmholtz Association, Berlin, Germany, 6. Laboratory of Neuroproteomics, Department of Biochemistry and Tissue Biology, Institute of Biology, University of Campinas (UNICAMP), São Paulo, Brazil, 7. Instituto Nacional de Biomarcadores em Neuropsiquiatria (INBION), Conselho Nacional de Desenvolvimento Científico e Tecnológico (CNPq), São Paulo, Brazil, 8. Núcleo de Neurociências e Comportamento, Departamento de Psicologia Experimental, Instituto de Psicologia, Universidade de São Paulo, São Paulo, Brazil

Corresponding authors:

Karina Griesi-Oliveira: karina.griesi@einstein.br

Maria Rita Passos-Bueno: [passos@ib.usp.br](mailto:passos@ib.usp.br)

*Patients ascertainment*

The patients enrolled in this study (n=6) were diagnosed by experienced psychiatrists following DSM-IV (Diagnostic and Statistical Manual of Mental Disorders, Fourth Edition) criteria. Patients were evaluated by the Childhood Autism Rating Scale (CARS) and Wechsler Intelligence Scale for Children (WISC) whenever possible (Supplementary Table S1). All the ASD individuals were males and negative for Fragile-X Syndrome, including five high functioning patients and one low functioning patient (IQ<70). The control sample consisted of 6 male individuals with no history of ASD or any other neurodevelopmental disorder diagnosis. This project has been approved by the Ethics Committee of the Instituto de Biociências – Universidade de São Paulo (protocol number 1.133.486), and was conducted in accordance with all the guidelines and legal regulations. After a complete description of the study, written informed consent was signed by the parents.

*array-CGH and exome sequencing*

Peripheral blood DNA was extracted from patients and their parents to investigate copy number variations (CNVs) and single nucleotide variants (SNVs). The presence of CNVs was evaluated by comparative genomic hybridization array (CGH-array) using an Agilent 4X 180K chip (Agilent Technologies, CA, USA), according to manufacturer's instructions. Arrays were scanned by Agilent High-Resolution Microarray scanner, and processed using Feature Extraction and Agilent Genomic Workbench softwares (Agilent Technologies). Only alterations comprising at least three consecutive oligonucleotide probes with an altered log2 signal ratio were considered as possible CNVs. The CNVs found were compared to the Database of Genomic Variants (<http://projects.tcag.ca/variation/>) and Database of Chromosomal Imbalance and Phenotype in Humans Using Ensembl Resources (DECIPHER; <http://decipher.sanger.ac.uk/>) to identify common and rare variants, as well as recurrence of variants in other autistic patients.

Exome sequencing libraries were generated using Nextera Rapid Capture Exome kit (Illumina, CA, USA) and sequenced on an Illumina HiSeq 2500 equipment to generate 150bp paired-end sequences, reaching 20x sequencing depth for at least 60% of base callings. Reads were aligned to the human genome (hg19) using Burrows-Wheeler Aligner^1^, variant calling was performed by Genome Analysis Toolkit^2^, with the recommended Best Practices protocol and standard thresholds, while ANNOVAR^3^ was used to annotate the variants. We sorted out loss-of-function variants (stop-gain, stop-loss, splicing) and missense variants (a) with a minor-allele-frequency (MAF) ≤ 0.01 in three different databases (1000 Genomes Project - <http://www.internationalgenome.org/>, NHLBI GO Exome Sequencing Project - <http://evs.gs.washington.edu/EVS/>, and ABraOM - <http://abraom.ib.usp.br/>; (b) with a MAF ≤0.05 in our in house NGS database, enriched by ASD individuals; (c) that were not in hypermutable genes; (d) with a sequencing depth ≥10; and (e) with an allele ratio ≤0.7 for heterozygous variants or ≤0.1 for homozygous variants. We then filtered only those variants present in genes expressed either in NPC or in neurons, with pLI≥0.8 (<http://exac.broadinstitute.org/>), for loss-of-function variants, or, in case of missense variants, with CADD-score ≥20. Finally, we reported only those variants present in the SFARI gene database^4^ (<https://gene.sfari.org/>) or in any of the co-expression modules found as ASD-associated.

*iPSC generation and characterization*

The iPSCs lines from each individual were generated from stem cells from human exfoliated deciduous teeth (SHED) established as described in Griesi-Oliveira et al., 2013^5^ and Griesi-Oliveira et al., 2015^6^. For iPSC generation, SHED were transduced with retroviral vectors containing *SOX2, c-Myc, OCT4* and *KLF4* genes, co-cultured with murine embryonic fibroblasts in human embryonic media (hES media) - DMEM/F12 (Thermo Fisher Scientific, MA, USA), 20% Knockout Serum Replacement (Thermo Fisher Scientific), 1% nonessential amino acids (Thermo Fisher Scientific) and 100 μM β- mercaptoethanol (Thermo Fisher Scientific), 30ng/ml FGF (Thermo Fisher Scientific) - and treated with 1 mM valproic acid (Sigma-Aldrich, MO, USA) for around 15 days until the emergence of iPSC colonies^6^. The colonies were then isolated and expanded in E8 media (Thermo Fisher Scientific) in matrigel coated dishes (BD Bioscience, NJ, USA). Two to three clones were generated from each sample and were tested for the presence of aneuploidies by Multiplex Ligation-dependent Probe Amplification (MLPA) (kits SALSA MLPA P036 and P070 - MRC-Holland, Amsterdam, The Netherlands). Only one clone from a control sample (F6119-1 cl.5) was found to have a mosaic trisomy of chromosome 12 and was excluded from further experiments. iPSC lines showed typical morphology and expression of pluripotency markers as attested by immunocytochemistry (Supplementary Fig. S1A,B). To attest their capacity of differentiation to the three germ layers, iPSC colonies were cultured in suspension in hES media without FGF for 7 days to induce random differentiation, and then, expression of endoderm, mesoderm and ectoderm markers was addressed by quantitative real-time PCR (Supplementary Fig. S1C).

*Neuronal differentiation*

After 4 days of iPSC plating, E8 media was replaced by 0.5x NB media - DMEM/F12, supplemented with 0.5x N2-supplement (Thermo Fisher Scientific) and 0.5x B27 serum (Thermo Fisher Scientific) - supplemented with 1uM of dorsomorphin (Tocris, Bristol, UK). After two days, colonies were gently detached and transferred to low attachment plates in order to form spheroids (Supplementary Fig. S1A). After two days, dorsomorphin was removed and the media was then supplemented with 20ng/ml of FGF and EGF (Peprotech, NJ, USA). Spheroids were cultured for 5 more days in this condition and then gently dissociated with accutase (Thermo Fisher Scientific) and plated in matrigel coated dishes. After 4 to 7 days, rosettes (Supplementary Fig. S1A) were manually isolated and transferred to plates coated with 10ug/ml of poli-ornitin (Sigma-Aldrich) and 5ug/ml of laminin (Thermo Fisher Scientific). The neuronal progenitor cells (NPCs) population obtained was expanded and stored for further experiments (Supplementary Fig. S1A). Some of the NPCs lineages were transduced with SYN::EGFP lenti-virus vector, a vector containing the sequence of green fluorescent protein under the control of the Synapsin gene promoter (kindly donated by Dr. Alysson Muotri). For neuron generation, NPC (either transduced with SYN::EGFP or not) were plated at low density (9,000cels/mm^2^) in 20ug/ml poli-ornitin and 10ug/ml laminin coated plates and media was changed in the next day to 1x NB, supplemented with 1uM of retinoic acid (Sigma-Aldrich). Cells were kept in this condition for 4 weeks with media changes every 2 or 3 days (Supplementary Fig. S1A).

*Immunocytochemistry*

Cells were fixed with 4% paraformaldehyde, washed 3x with PBS, incubated for 40 minutes in blocking buffer (5% donkey serum, 0.1% Triton-X in PBS), and then incubated overnight at 4oC with primary antibody. In the next day, cells were washed 3x with PBS and incubated with secondary antibodies, washed 3x again and mounted with Vectashield with DAPI. Images for iPSC, NPC and neuron characterization were taken in an Olympus IX51 confocal microscope. Primary antibodies used: anti-SOX2 (1:100, Millipore, MA, USA), anti-Nestina (1:250, Millipore), anti-CTIP2 (1:500, Millipore), anti-MAP2 (1:500, Millipore). Secondary antibodies: Alexa Fluor 488 donkey and Alexa Fluor 594 donkey anti-mouse at 1:250 dilution (Thermo Scientific).

*Cell sorting*

GFP-expressing neurons were sorted out from heterogeneous populations of cells generated after 4 weeks of differentiation from NPCs. For that, cells were detached from plates after 30 minutes incubation with Tryple express (Thermo Fisher Scientific), centrifuged and passed through a 40um filter in order to eliminate cell clumps. Small cells with high GFP expression were selected by fluorescence-activated cell sorting in a BD Bioscience FACSAria II Machine, while large cells, although GFP-positive, were discarded (Supplementary Fig. S2).

*RNA sequencing and normalization of expression data*

RNA was extracted using NucleoSpin RNA extraction kit (Macherey-Nagel, Germany) from NPCs (29 cell lines) and from heterogeneous populations of neuronal cells (16 cell lines) or sorted GFP-expressing neurons (7 cell lines) obtained after 4 weeks of neuronal differentiation (Supplementary Table S2). RNA integrity and quality were checked by capillary electrophoresis in a 2100 BioAnalyzer equipment (Agilent Technologies, CA, USA) and quantified in a Nanodrop 2000 spectrophotometer (Thermo Fisher Scientific). 1000ng of RNA was used for library preparation using the Truseq RNA Sample Preparation kit (Illumina, CA, USA) and then sequenced on a Hiseq 2500 (Illumina) to generate 100bp paired-end sequences. 20 million reads were randomly selected from each sample for gene expression analyses. Sequences were aligned to the reference human genome (hg19) using TopHat2 ^7^ and read counts per gene were summarized using HTSeq ^8^. Data is available at Gene Expression Omnibus website under the accession number GSE142670. Fragments per million kilobase (FPKM) were calculated and only genes with FPKM≥1 in more than half of the samples (calculated separately for NPC and neurons) were considered as expressed and retained for analysis (NPC:13818 genes; neurons: 15026 genes). *In silico* deconvolution of gene expression data^9^ was carried out to estimate neuronal and astrocyte proportions for each differentiated sample, using the DeconRNASeq algorithm with default parameters^10^. Gene expression data from pure cultures of human neurons and astrocytes was acquired from the FANTOM5 consortium^11^. Only protein coding genes were used for deconvolution analyses. Estimated proportions are listed in Supplementary Table S2. Differentiated samples with an estimated proportion of neurons lower than 50% were excluded, leaving a total number of 19 neuron samples (7/7 GFP-sorted samples, and 11/16 samples of heterogeneous populations of neuronal cells) for further analyses (Supplementary Table S2). To correct for unwanted variation, including differences in cell type proportions across samples, expression data were normalized using RUVseq^12^. For normalization, RUVseq algorithm uses two parameters, defined by the user: a set of control genes (i.e., a set of genes that it is believed not to be influenced by the variable of interest); and a number k of unwanted variation factors. To select the control genes, a first-pass differential expression analysis comparing ASD to control samples was performed using DESeq2^13^, and 9 sets of not differentially expressed genes were selected varying the p-value from 0.1 to 0.9, by 0.1 increases. Each of these sets was then overlapped with a list of housekeeping genes obtained from Eisenberg & Levanon, 2013^14^, so only those not differentially expressed genes that were also identified as housekeeping genes by these authors were kept in the final sets of control genes. For each of these 9 sets, the parameter k was varied from 1 to 5. The final parameters (set of control genes and parameter k) considered for normalization of data were determined so that there was no significant correlation between estimated neuronal proportions and the first two principal components of gene expression data (Supplementary Table S3), while leading to the best clustering of replicates (Supplementary Figure S3). Expression values used for sample clustering were based on variance stabilizing transformation, which was calculated using DESeq2. Thus, for NPC, the selected p-value threshold for control genes list was ≥0.3 and k=2, while, for neurons we used a p-value threshold of ≥0.4 and k=4. RUVseq normalized counts were then used for all the subsequent analysis.

*Prediction of regional identity and developmental period*

To estimate the maturity of the cells and their regional identity, transcriptome profiles of each of NPC and neuron samples were compared to the transcriptome data of brain samples available at BrainSpan Atlas (www.brainspan.org), whose ages vary between 4 post-conceptional weeks until 60 years-old, using a machine learning approach developed by Stein et al., 2014^15^ (<https://context.semel.ucla.edu/>).

*Differential expression and Weighted gene correlation network analysis*

Differentially expressed genes were identified using *dream* algorithm from variationPartition package^16^. Functional enrichment analysis was conducted using cameraPR, to identify if genes related to a particular function are highly ranked relative to other genes in terms of differential expression^17^. Weighted-gene co-expression network analysis was performed using WGCNA package from R^18^. Co-expression networks were constructed separately for NPC and Neurons, following the same analysis workflow for both cell types, except for specific parameters when noted. We selected the top ~10.000 genes with the highest variance among those genes considered as expressed (see above). Network construction and module assignment were obtained using the function blockwiseModule, with the following parameters: power=11 (NPC) or 16 (Neurons), networkType = "signed", minModuleSize=50 (NPC) or 150 (Neurons), mergeCutHeight=0.15, verbose=6, minKMEtoStay = 0.5, nThreads=24 and maxBlockSize=20000. Module M0 (grey) was set to comprise the genes that could not be clustered into any specific module. For each module, gene expression levels of each sample were summarized in an eigengene value which was then used to assess the correlation of a module to disease status, the batch of library preparation or the neuronal proportion within the samples. Functional annotation analysis of the modules was performed using the Database for Annotation, Visualization and Integrated Discovery 6.8 (DAVID - https://david-d.ncifcrf.gov/) and Ingenuity Pathway Analysis Software (http://www.ingenuity. com/). Protein-protein enrichment test was performed using STRING (<https://string-db.org/cgi/input.pl>). Module preservation analysis^19^ was conducted using *modulePreservation* function from WGCNA, comparing our data to BrainSpan fetal brain samples. Data and analysis code are available at: <https://github.com/griesik/ASDiPSCTranscriptome.git>

Neuronal morphological analysis

Twenty thousand cell-sorted GFP-expressing neurons were plated with 50.000 cells of a heterogeneous population of neuronal cells differentiated from NPC not transduced with SYN::EGFP either from the same individual (non-mixed condition) or from an individual from the opposite group (mixed condition), in wells from an 8-well chamber slide. Seventy-two hours after plating, cells were fixed and immunostained for green fluorescent protein, as described above. Images were taken at 20x with InCell Analyzer 2200 microscope (GE Healthcare, Chicago IL, USA) and morphological analysis was conducted using Neurphology plugin from ImageJ. Measures obtained were normalized by the number of nuclei in each image and statistical analysis was performed using geepack package from R^20^.

*Mass spectometry-based proteomics*

Protein was extracted from NPC of 6 clones (3 patients, 3 controls). Protein extracts were trypsinized at a ratio of 1:80 (trypsin: total protein). Resulting peptides were lyophilized and further dissolved in an aqueous solution of 0.1% formic acid. These were injected into two-dimensional, reverse-phase liquid chromatography using an Acquity UPLC M-Class System (Waters Corporation, Milford, MA) coupled to a Synapt G2-Si mass spectrometer (Waters Corporation, Milford, MA). Specifics about data acquisition are described in detail in Cassoli et
al 2017^21^. Briefly, 3 μg of peptides were automatically loaded into a M-Class BEH C18 Column (130 Å, 5 μm, 300 μm × 50 mm, Waters Corporation, Milford, MA). First dimension fractionation was done using discontinuous steps of acetonitrile (11%,
20%, and 50%). After each step, eluted peptides undergone to second-dimension separation, in a nanoACQUITY UPLC HSS T3 Column (100 Å, 1.8 μm, 75 μm × 150 mm, Waters Corporation, Milford, MA). Peptide were eluted using an 36 minutes acetonitrile gradient from 7% to 40%
(v/v) at a flow rate of 0.4 μL/min. These were electrosprayed in positive ion mode online into a Synapt G2-Si. The mass spectrometer acquired in data-independent acquisition mode (DIA) with ion-mobility separation (Ref: PMID: 27896757). A NanoLock Spray (Waters, Manchester, UK) was used to deliver [Glu1]-Fibrinopeptide B human (Glu-Fib) as an internal standard.

*Proteomics data analysis*

Mass spectrometric raw data was processed with Progenesis®QI version 2.1 (Waters) and proteins were identified. Quantitative data was processed using dedicated algorithms and searched against the Uniprot human proteomics database, with the default parameters for ion
accounting and quantitation. More details about data search can be found in Cassoli et al. 2017^21^. Differentially expressed proteins were identified by anova test (p<0.05). Genes that regulate a significant number of genes from M_Neu_1-turquoise or M_Neu_18-purple (i.e., upstream regulators) were identified using Ingenuity Pathways Analysis (IPA) software (<http://www.ingenuity.com/>) and these lists were then confronted against the differentially expressed proteins (DEP) list in order to check if any predicted upstream regulator was also a DEP. Spearman correlation between the NPC protein expression levels and M_NPC_10-blue, M_Neu_1-turquoise and M_Neu_18-purple module eigengenes of the same samples included in the proteomic analysis was calculated in order to find proteins whose expression levels were concomitantly correlated to both M_NPC_10-blue and M_Neu_1-turquoise or M_NPC_10-blue and M_Neu_18-purple. The interactome of each of these proteins were retrieved from IPA database and then tested for enrichment for M_Neu_1-turquoise or M_Neu_18-purple genes using a two-sided Fisher’s exact test calculated by the R function fisher.test, corrected for false discovery rate.

*Variants and Sfari genes enrichment analysis*

A list of rare *de novo* exonic variants found in ASD individuals and their siblings was obtained from Kosmick et al., 2016^22^ (a compilation of the original papers from de Rubeis et al., 2016^23^; Iossifov et al., 2012^24^). For this analysis, we only considered missense variants with a CADD-score ≥20 (ref) and loss-of-function variants with pLi≥0.8 that are not present in an Exac database population sample which psychiatric patients were excluded from (Exac_nonpsy). ASD genes were compiled from Sfari database (as by January 2019, restricted to categories 1 to 4) and ID genes was obtained from ID genetics database^25^ (<http://www.ccgenomics.cn/IDGenetics/>). For ID genes, we only considered those listed as “known genes” plus those listed as “candidate genes” that had “intellectual disability” or “global developmental delay” as descriptors in the associated phenotypes. Only the protein-coding genes from the co-expression modules were considered for gene set enrichment, which was performed using a two-sided Fisher’s exact test calculated by the R function *fisher.test*, corrected for false discovery rate.

*Module overlap analysis*

The function *userListEnrichment* from WGCNA was used to evaluate the overlap between the modules identified in our study and the modules identified by others^26–34^. This was run separately for NPC modules and neuron modules. To build the “consensus module” (the list of genes with strong evidence of being part of the module of synapse genes associated with ASD), we considered all the modules from the studies that used either fetal/neonatal brain samples or iPSC-derived neurons that presented a significant overlap with M_Neu_1-turquoise, namely: modules M13, M16 and M17 from Parikshak et al., 2013; modules brown, blue, pink and tan from Mariani et al., 2015; and module lightyellow from Schafer et al., 2019. Although module M19^d35^ from De Rosa et al., 2018, a study conducted with iPSC-derived neurons, also presented a significant overlap with M_Neu_1-turquoise, it was excluded from the generation of the consensus module because data of the entire set of expressed genes in this study were unavailable, which would be necessary to set the proper gene background to calculate Sfari genes enrichment in the consensus module. Sfari genes enrichment within each of the modules from individual studies and within consensus module was calculated considering the whole list of Sfari genes (“Sfari all”; not excluding ID genes) using the R function *fisher.test*, corrected for false discovery rate. Venn-diagram was generated with the public available tool <http://bioinformatics.psb.ugent.be/webtools/Venn/>.

**Supplementary references**

1. Li H, Durbin R. Fast and accurate short read alignment with Burrows – Wheeler transform. Bioinformatics. 2009;25(14):1754–60.

2. Mckenna A, Hanna M, Banks E, Sivachenko A, Cibulskis K, Kernytsky A, et al. The Genome Analysis Toolkit : A MapReduce framework for analyzing next-generation DNA sequencing data. Genome Res. 2010;20(9):1297–303.

3. Wang K, Li M, Hakonarson H. ANNOVAR : functional annotation of genetic variants from high-throughput sequencing data. Nucleic Acids Res. 2010;38(16):e164.

4. Abrahams BS, Arking DE, Campbell DB, Mefford HC, Morrow EM, Weiss LA, et al. SFARI Gene 2.0: a community-driven knowledgebase for the autism spectrum disorders (ASDs). Mol Autism. 2013;4(1):36.

5. Griesi-Oliveira K, Sunaga DY, Alvizi L, Vadasz E, Passos-Bueno MR. Stem cells as a good tool to investigate dysregulated biological systems in autism spectrum disorders. Autism Res. 2013;6(5):354–61.

6. Griesi-Oliveira K, Acab A, Gupta AR, Sunaga DY, Chailangkarn T, Nicol X, et al. Modeling non-syndromic autism and the impact of TRPC6 disruption in human neurons. Mol Psychiatry. 2015;20(11):1350–65.

7. Trapnell C, Pachter L, Salzberg SL. TopHat : discovering splice junctions with RNA-Seq. Bioinformatics. 2009;25(9):1105–11.

8. Anders S, Pyl PT, Huber W. Genome analysis HTSeq — a Python framework to work with high-throughput sequencing data. Bioinformatics. 2015;31(2):166–9.

9. Hoffman GE, Hartley BJ, Flaherty E, Ladran I, Gochman P, Ruderfer DM, et al. Transcriptional signatures of schizophrenia in hiPSC-derived NPCs and neurons are concordant with post-mortem adult brains. Nat Commun. 2017;8(1):2225.

10. Gong T, Szustakowski JD. DeconRNASeq : a statistical framework for deconvolution of heterogeneous tissue samples based on mRNA-Seq data. Bioinformatics. 2013;29(8):1083–5.

11. FANTOM Consortium and the RIKEN PMI and CLST (DGT), Forrest AR KH, Rehli M, Baillie JK de HM. A promoter-level mammalian expression atlas. Nature. 2014;507(7493):462–70.

12. Risso D, Ngai J, Speed TP, Dudoit S. Normalization of RNA-seq data using factor analysis of control genes or samples. Nat Biotechnol. 2014;32(9):896–902.

13. Love MI, Huber W, Anders S. Moderated estimation of fold change and dispersion for RNA-seq data with DESeq2. Genome Biol. 2014;15:550.

14. Eisenberg E, Levanon EY. Human housekeeping genes, revisited. Trends Genet. 2013;29(10):569–74.

15. Stein JL, de la Torre-Ubieta L, Tian Y, Parikshak NN, Hernández IA, Marchetto MC, et al. A quantitative framework to evaluate modeling of cortical development by neural stem cells. Neuron. 2014;83(1):69–86.

16. Hoffman GE, Roussos P. dream: Powerful differential expression analysis for repeated measures designs. bioRxiv. 2018;432567. Available from: https://www.biorxiv.org/content/10.1101/432567v1

17. Wu D, Smyth GK. Camera: A competitive gene set test accounting for inter-gene correlation. Nucleic Acids Res. 2012;40(17):e133.

18. Langfelder P, Horvath S. WGCNA : an R package for weighted correlation network analysis. BMC Bioinformatics. 2008;9:559.

19. Langfelder P, Luo R, Oldham MC, Horvath S. Is My Network Module Preserved and Reproducible? PLoS Comput Biol. 2011;7(1):e1001057.

20. Halekoh U, Højsgaard S, Yan J. The R package geepack for generalized estimating equations. J Stat Softw. 2006;15(2):1–11.

21. Cassoli JS, Brand C, Santana AG, Souza GHMF, Martins-de-souza D. Ion Mobility-Enhanced Data-Independent Acquisitions Enable a Deep Proteomic Landscape of Oligodendrocytes. Proteomics. 2017;17(21).

22. Kosmicki JA, Samocha KE, Howrigan DP, Sanders SJ, Slowikowski K, Lek M, et al. Refining the role of de novo protein-truncating variants in neurodevelopmental disorders by using population reference samples. Nat Genet. 2017;49(4):504–10.

23. De Rubeis S. Synaptic, transcriptional and chromatin genes disrupted in autism. Nature. 2014;515(7526):209–15.

24. Iossifov I, Ronemus M, Levy D, Wang Z, Hakker I, Rosenbaum J, et al. De Novo Gene Disruptions in Children on the Autistic Spectrum. Neuron. 2012;74(2):285–99.

25. Chen C, Chen D, Xue H, Liu X, Zhang T, Tang S, et al. IDGenetics : a comprehensive database for genes and mutations of intellectual disability related disorders. Neurosci Lett. 2018;685:96–101.

26. Voineagu I, Wang X, Johnston P, Lowe JK, Tian Y, Horvath S, et al. Transcriptomic analysis of autistic brain reveals convergent molecular pathology. Nature. 2011;474(7351):380–4.

27. Tylee DS, Hess JL, Quinn TP, Barve R, Huang H, Zhang-james Y, et al. Blood Transcriptomic Comparison of Individuals With and Without Autism Spectrum Disorder : A Combined-Samples Mega-Analysis. Am J Med Genet Part B Neuropsychiatr Genet. 2017;174(3):181–201.

28. Parikshak NN, Swarup V, Belgard TG, Irimia M, Ramaswami G, Gandal MJ, et al. Genome-wide changes in lncRNA, splicing, and regional gene expression patterns in autism. Nature. 2016;540(7633):423–7.

29. Gupta S, Ellis SE, Ashar FN, Moes A, Bader JS, Zhan J, et al. Transcriptome analysis reveals dysregulation of innate immune response genes and neuronal activity-dependent genes in autism. Nat Commun. 2014;5:5748.

30. Mariani J, Coppola G, Zhang P, Pelphrey KA, Howe R, Vaccarino FM, et al. FOXG1-Dependent Dysregulation of GABA/ Glutamate Neuron Differentiation in Autism Spectrum Disorders Graphical. Cell. 2015;162(2):375–90.

31. Parikshak NN, Luo R, Zhang A, Won H, Lowe JK, Chandran V, et al. Integrative Functional Genomic Analyses Implicate Specific Molecular Pathways and Circuits in Autism. Cell. 2013;155(5):1008–21.

32. Gandal MJ, Haney JR, Parikshak NN, Leppa V, Ramaswami G, Hartl C, et al. Shared molecular neuropathology across major psychiatric disorders parallels polygenic overlap. Science. 2018;359:693–7.

33. Derosa BA, Hokayem J El, Artimovich E, Garcia-serje C, Phillips W, Booven D Van, et al. Convergent Pathways in Idiopathic Autism Revealed by Time Course Transcriptomic Analysis of Patient- Derived Neurons. Sci Rep. 2018;8(1):8423.

34. Schafer ST, Paquola ACM, Stern S, Gosselin D, Ku M, Pena M, et al. Pathological priming causes developmental gene network heterochronicity in autistic subject-derived neurons. Nat Neurosci. 2019;22(2):243–55.
