# Supplementary figures and images for: Transcriptome of iPSC-derived neuronal cells reveals a module of co-expressed genes consistently associated with autism spectrum disorder
Source: Mol Psychiatry. 2020 Feb 14;26(5):1589–605. doi: 10.1038/s41380-020-0669-9 (PMC8159745; doi:10.1038/s41380-020-0669-9)

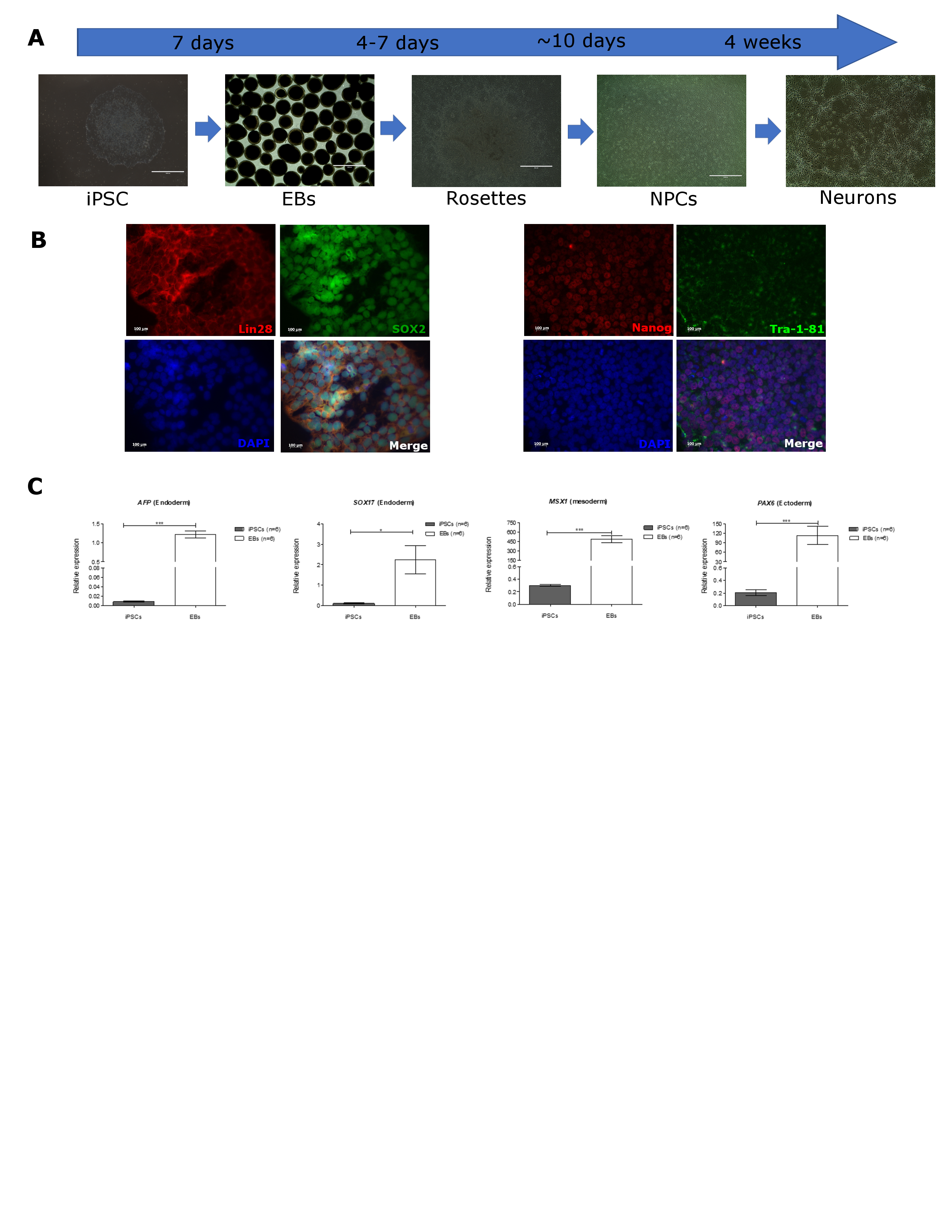

Supplement: Supplementary file 2 — Supp Fig. S1 [file 41380_2020_669_MOESM2_ESM.tif]

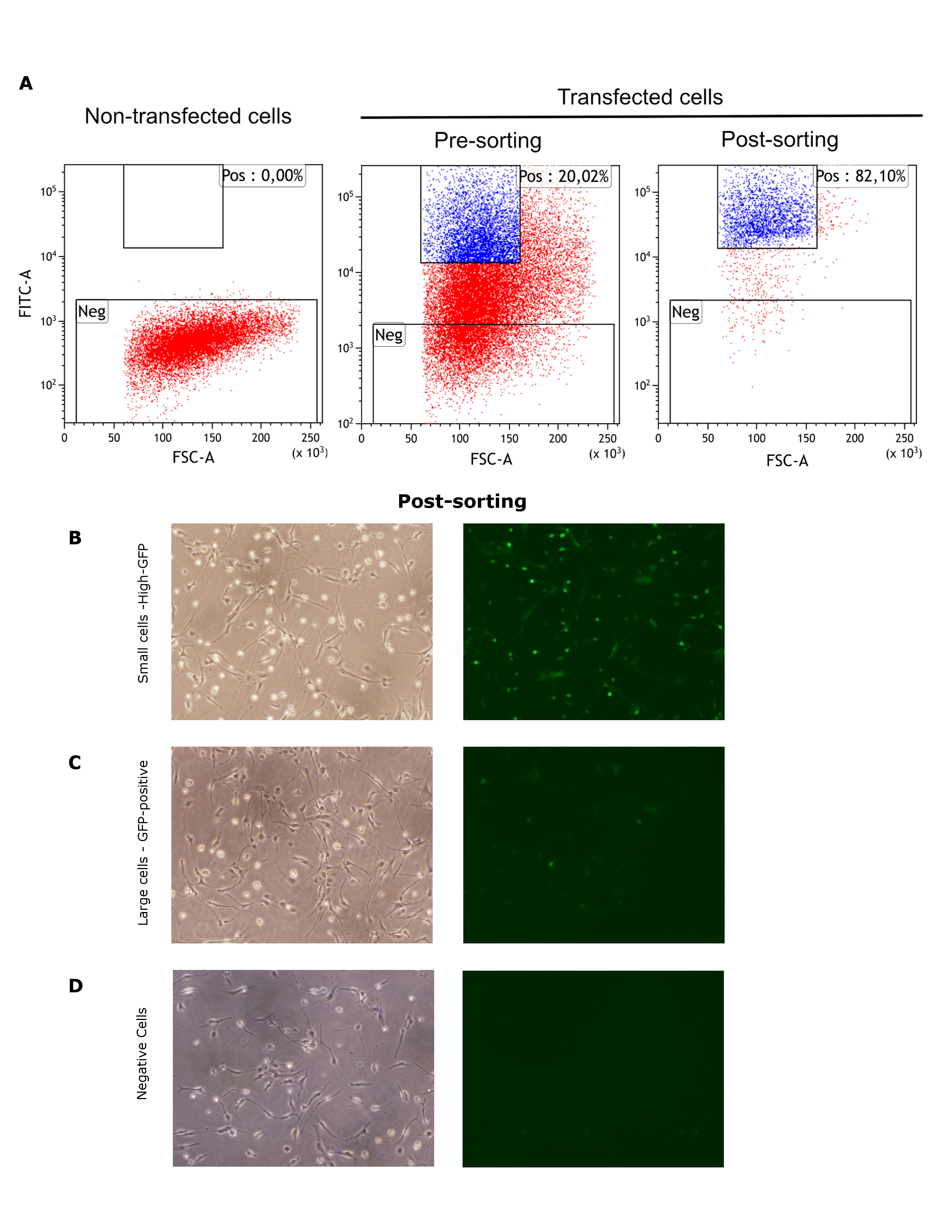

Supplement: Supplementary file 3 — Supp Fig. S2 [file 41380_2020_669_MOESM3_ESM.tif]

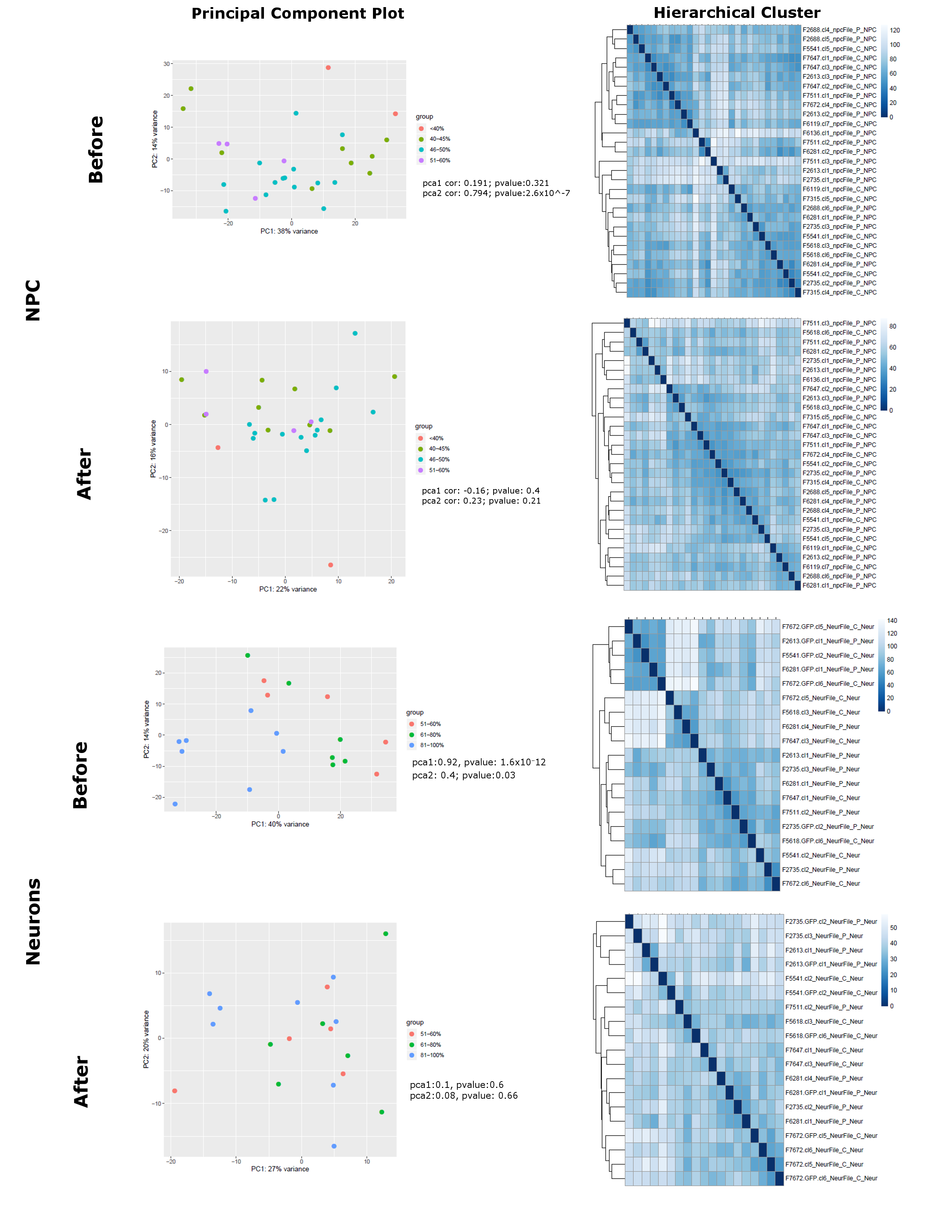

Supplement: Supplementary file 4 — Supp Fig. S3 [file 41380_2020_669_MOESM4_ESM.tif]
