# Supplementary material for: Transcriptome of iPSC-derived neuronal cells reveals a module of co-expressed genes consistently associated with autism spectrum disorder
Source: Mol Psychiatry. 2020 Feb 14;26(5):1589–605. doi: 10.1038/s41380-020-0669-9 (PMC8159745; doi:10.1038/s41380-020-0669-9)
Supplement: Supplementary file 18 — Supplementary Figure Legends [file 41380_2020_669_MOESM18_ESM.docx]

**Supplementary Figure Legends**

**Supplementary Figure S1. Reprograming, neuronal differentiation and molecular characterization of iPSC lines.** (A) Timeline for cell reprogramming and neuronal differentiation, showing morphological characteristics of cells in each step of the protocol. (B) Immunocytochemistry for molecular characterization of the iPSC: cells present the expression of characteristic pluripotency markers such as LIN28, NANOG, SOX2 and TRA-1-81. (C) Relative quantification of endoderm, mesoderm and ectoderm gene markers for iPSC and cells derived from iPSC after random differentiation, showing the capacity of the iPSC to be differentiated in cells from the three germ layers.

**Supplementary Figure S2. Cell sorting for neuron enrichment.**  (A) Gate strategy used to sort the cells with the highest green fluorescence while selecting for those with smaller cell body. Morphology and GFP expression of (B) small cells with high GFP expression (positive population), (C) large cells with GFP expression and (D) cells with no GFP expression (negative population).

**Supplementary Figure S3. Normalization of expression data.** Principal component analysis and hierarchical clustering of NPC and neurons samples before and after normalization with RUVseq. The final parameters (set of control genes and parameter k) considered for normalization of data were determined so that there was no significant correlation between estimated neuronal proportions and the first two principal components of gene expression data, while leading to the best clustering of replicates.
